# Discovery of selective activators of PRC2 mutant EED-I363M

**DOI:** 10.1038/s41598-019-43005-z

**Published:** 2019-04-25

**Authors:** Junghyun L. Suh, Kimberly D. Barnash, Tigran M. Abramyan, Fengling Li, Juliana The, Isabelle A. Engelberg, Masoud Vedadi, Peter J. Brown, Dmitri B. Kireev, Cheryl H. Arrowsmith, Lindsey I. James, Stephen V. Frye

**Affiliations:** 1 Center for Integrative Chemical Biology and Drug Discovery, Division of Chemical Biology and Medicinal Chemistry, UNC Eshelman School of Pharmacy, University of North Carolina at Chapel Hill, Chapel Hill, North Carolina 27599 USA; 2 Present Address: Foghorn Therapeutics, Cambridge, MA 02142 USA; 3 Structural Genomics Consortium, University of Toronto, Toronto, Ontario, M5G 1L7 Canada; 4 Princess Margaret Cancer Centre and Department of Medical Biophysics, University of Toronto, Toronto, Ontario, M5G 2M9 Canada

**Keywords:** Computational chemistry, Myelodysplastic syndrome

## Abstract

Many common disease-causing mutations result in loss-of-function (LOF) of the proteins in which they occur. LOF mutations have proven recalcitrant to pharmacologic intervention, presenting a challenge for the development of targeted therapeutics. Polycomb repressive complex 2 (PRC2), which contains core subunits (EZH2, EED, and SUZ12), regulates gene activity by trimethylation of histone 3 lysine 27. The dysregulation of PRC2 catalytic activity by mutations has been implicated in cancer and other diseases. Among the mutations that cause PRC2 malfunction, an I363M LOF mutation of EED has been identified in myeloid disorders, where it prevents allosteric activation of EZH2 catalysis. We describe structure-based design and computational simulations of ligands created to ameliorate this LOF. Notably, these compounds selectively stimulate the catalytic activity of PRC2-EED-I363M over wildtype-PRC2. Overall, this work demonstrates the feasibility of developing targeted therapeutics for PRC2-EED-I363M that act as allosteric agonists, potentially correcting this LOF mutant phenotype.

## Introduction

The discovery of small molecules capable of targeting mutated and overexpressed proteins that act as drivers of disease ushered in the modern era of oncology drug discovery^1^. Unfortunately, many disease-causing mutations are difficult to target directly because they result from loss-of-function (LOF) mutations in the proteins in which they occur^2^. LOF mutant proteins are often misfolded, destabilized, and otherwise not biochemically active, making them more difficult to ‘rescue’ therapeutically compared to inhibiting an intact, but over-active protein (the targets of the vast majority of drugs). While approaches relying on protein replacement and gene therapy can correct some of these deficits, they suffer from high-costs, require parenteral routes of administration, and are difficult to apply in less developed locations. Therefore, discovery of small molecule pharmacologic methods to correct for LOF mutations is critical to expanding the applicability of genetically targeted approaches in the treatment of human disease.

The 400 or so proteins involved in chromatin structure and epigenetic gene regulation are frequently subject to mutations associated with developmental disorders and cancer^3,4^. Changes in chromatin structure serve to regulate the genome by controlling access to the underlying DNA. The deposition, recognition, and removal of post-translational modifications (PTM) on histones is one mechanism by which chromatin structure can be regulated, and the dysregulation of these PTMs has been implicated in tumorigenesis and various diseases^5–7^.

Polycomb group (PcG) complexes, PRC1 and PRC2, are critical regulators of transcriptional repression *via* their ability to modify chromatin structure at target genes. Consequently, they play key roles in development, stem cell self-renewal, differentiation, and disease^7–9^. PRC2 is composed of three essential subunits including EZH1/2, EED, SUZ12, while a fourth subunit, RbAp46/48, is thought to be necessary for full methyltransferase activity. Importantly, the catalytic SET domain of EZH1/2 is known to adopt an inactive conformation and association with EED and SUZ12 is required for activation^10–14^. EED is a methyl-lysine (Kme) reader protein of the WD40 family. Through the binding of its aromatic cage to H3K27me3, the catalytic product of PRC2, as well as JARID2, a PRC2 accessory protein methylated at lysine 116 (K116me3), EED functionally stimulates PRC2 activity. Recent structural studies revealed that the ability of EED to allosterically activate EZH2 depends on its binding to these methylated substrates, which serves to stabilize the active conformation of EZH2. Specifically, the stimulation-responsive motif (SRM) helix of EZH2 exhibits a disorder-to-order conformational transition upon binding of EED to a methylated peptide^10,11,14–17^.

Several mutations of PRC2 subunits have been reported which disrupt normal PRC2 function, resulting in diseases such as lymphoma, prostate cancer, and Weaver syndrome^9,18–22^. Gain-of-function (GOF) mutations within the catalytic SET domain of EZH2 have been implicated in several types of lymphoma. These mutations increase the trimethylase activity of the enzyme thereby increasing the levels of trimethyl lysine 27 (H3K27me3) in cells and aberrantly repressing gene expression^19,23–26^. A number of small-molecule inhibitors targeting either the catalytic SET domain of EZH2 or the EED-methyl-lysine interface have been developed to antagonize this upregulated PRC2 activity^23,27–29^. Among them, A-395 and EED226 are recently reported PRC2 allosteric antagonists that bind to the H3K27me3 binding site on the beta-propeller WD40 domain of EED by remodeling the EED binding pocket, preventing stabilization of the SRM helix and subsequent PRC2 catalytic activation^28,29^. In common with other small molecule targeted therapeutics, these agents all serve to decrease the activity of a GOF mutation.

Mutations also occur outside the PRC2 catalytic domain: EED-I363M, which is a LOF mutation, has been identified in patients with myelodysplastic syndrome (MDS) and related diseases. This mutation leads to increased susceptibility to myeloid cancers by impairing EED binding to H3K27me3, thereby abrogating allosteric activation of PRC2 catalytic activity and suppressing propagation of H3K27me3 repressive histone marks^20,30^. I363 is located adjacent to the EED methyl-lysine binding pocket, yet a detailed mechanistic understanding of how EED-I363M prevents H3K27me3 binding remains elusive. Furthermore, EED-I363M is expressed at similar levels to that of wildtype EED and is incorporated into PRC2 in cells^20,30^, making it a potential target for a mutant-selective agonist that could re-activate the EED-I363M mutant PRC2 enzyme. Consequently, we sought to pursue the development of ligands that bind EED-I363M, allosterically induce the active conformation of EZH2, and activate PRC2 catalysis in a similar fashion to the cognate ligand with wildtype PRC2, thereby correcting this LOF mutation and restoring normal levels of H3K27 methylation.

Historically, the ability to pharmacologically reverse the functional consequences of disease-causing, LOF mutations has been a challenge. In this study, we combined structure-based design and computational simulations to create mutant-selective allosteric agonists of PRC2-EED-I363M. Using previously reported WT-EED allosteric antagonists as a template, we were able to rationally modify these inhibitors to create mutant-selective activators, which were characterized in a PRC2 catalytic activity assay. Computational simulations further revealed the structural details of ligand binding and a rationale for their mechanism of action. Finally, we anticipate that these proof-of-concept tool compounds will inspire the development of more drug-like EED-I363M activators in an effort to restore PRC2 function in disease relevant settings, such as MDS^20,30^.

## Results

### Design and synthesis of peptidomimetic allosteric activators

**Figure 1:**
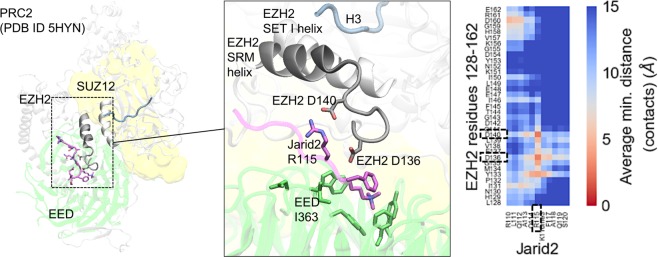
Structure and dynamics of PRC2 activation. Crystal structures of PRC2^10^ (left and zoomed in Jarid2 peptide binding site) and supporting MD simulations reveal the mechanism of PRC2 activation: upon EED (green) binding Jarid2 R115 (magenta) forms a salt bridge with D140 (and to a lesser extend with D136) on EZH2 loop stabilizing the conformation of EZH2 SRM helix (gray) which then in turn stabilizes EZH2 SET-I helix (white) of the catalytic binding site for substrate H3K27 (blue). The heatmap plot represents the average minimum residue-residue distances (contacts) between EZH2 and Jarid2 peptide highlighting the importance of the salt bridge, as determined from cumulative 2.5 µs MD sampling (SI Fig. S2). For clearer representation the labels on EED aromatic cage residues (F97, Y148, W364, Y365) and D362 as well as Jarid2’s K116me3 and F117 are hidden.

Recent structural and molecular studies have provided critical insight into the mechanism by which PRC2 activity is regulated by EED binding to JARID2 K116me3 (or H3K27me3) (PDB ID: 5HYN) (Fig. 1)^10,15^. In brief, methylated JARID2 binds EED and is then sandwiched between EED and EZH2 stabilizing EZH2’s SRM helix (residues 143–153) adjacent to the catalytic SET domain. The SRM helix then binds to the i-SET domain, decreasing its occupancy of the substrate-binding channel, thus maintaining EZH2 in its catalytically active conformation. Based on this mechanism, we aimed to design a synthetic activator that would both bind the Kme reader pocket of EED-I363M and retain the ability of JARID2 to stabilize the SRM helix of EZH2.

We previously reported peptidomimetic ligands of EED including UNC4859 and UNC5114 which are JARID2- and H3K27me3-competitive and function as allosteric inhibitors of EZH2 catalytic activity (Supporting Information (SI) Fig. S1A,B)^31^. These ligands display a significant affinity for wildtype EED (*K*_*d*_ ~ 0.7–1.1 µM) and hence represent suitable structural templates onto which JARID2-like features can be installed to recapitulate key stabilizing contacts with the SRM helix. Importantly, these peptidomimetic inhibitors adopt a binding mode that is analogous to the endogenous EED ligands making them well-suited starting points for the discovery of PRC2 activators. In contrast, the recently reported potent and selective small molecule EED inhibitors, A-395 and EED226^28,29^, effectively restructure the EED binding pocket and the top face of the WD40 beta barrel, which would make transforming these inhibitors into PRC2 activators less plausible.

**Figure 2:**
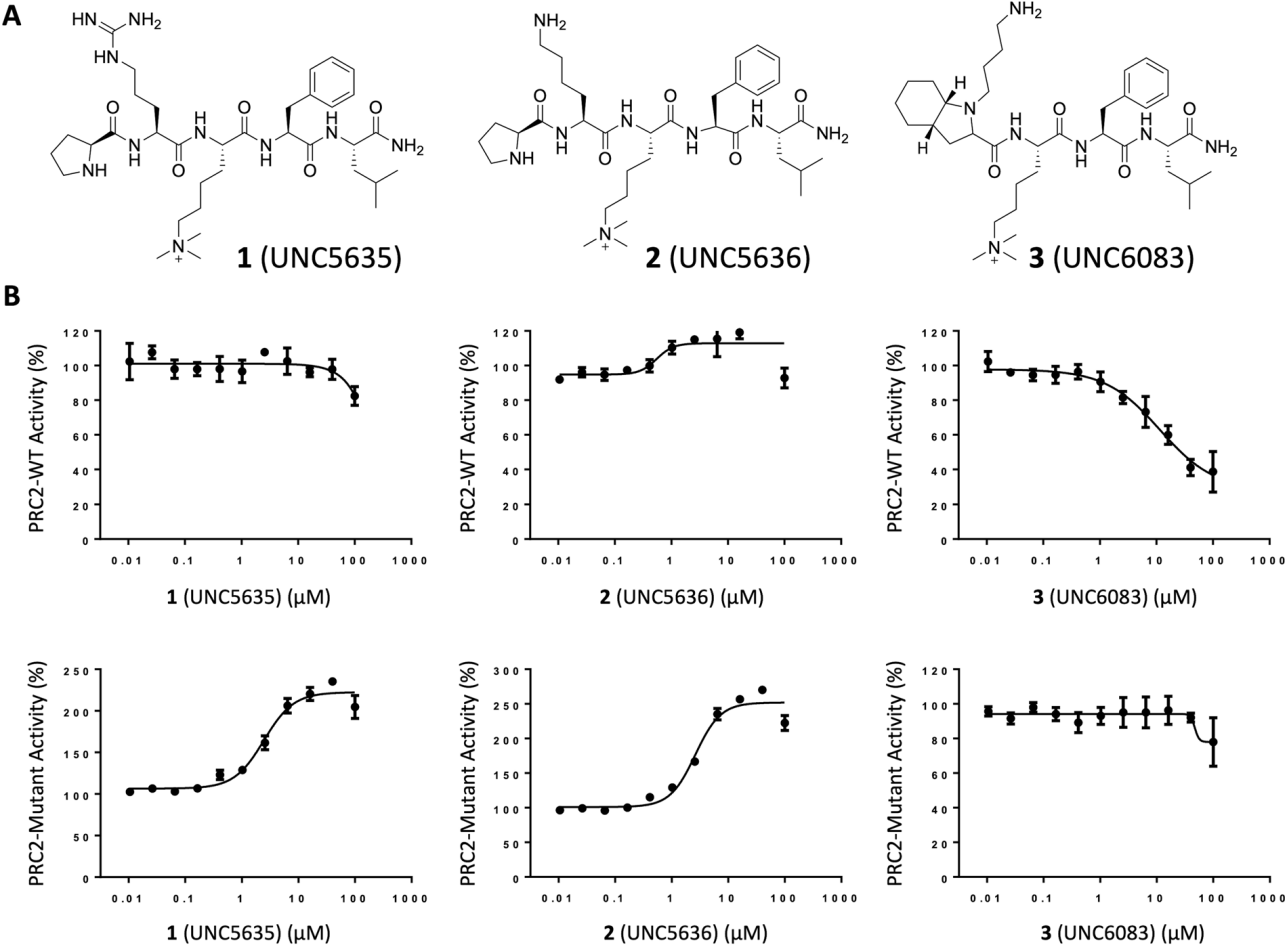
Selective activation of PRC2-EED-I363M mutant. Effect of (**A**) compound 1 (UNC5635), 2 (UNC5636) and 3 (UNC6083) on (**B**) methyltransferase activity of PRC2-WT and PRC2-EED-I363M mutant was assessed with different concentrations of compounds. For each complex, the activity of the enzyme in the presence of each compound has been normalized to the activity of that complex. All experiments were performed in triplicate using H3 peptide as a substrate as decribed in methods.

We first sought to identify key structural features of JARID2 that are responsible for its activator function. Our MD simulations indicate that the main interactions between JARID2 and EZH2 include salt bridges between R115 of JARID2 and D140 and, to a lesser extent, D136 of EZH2 as determined by calculating the average minimum residue-residue distances (Fig. 1 and SI Fig. S2A,B). These interactions were persistent in WT PRC2-EED and appeared to be the major factor in stabilizing the SRM helix. Both interactions were less frequent in mutant PRC2 containing EED-I363M, eventually leading to the SRM helix unfolding (SI Fig. S2C,D). Based on these insights, two series of potential activators were designed using UNC4859 and UNC5114 as structural templates (Fig. 2 and SI Fig. S3)^31^. First, ligands **1** (UNC5635) and **2** (UNC5636) were obtained by replacing the serine residue of UNC4859 with arginine and lysine, respectively, with the intent that these charged residues could similarly engage D140 and D136 of the SRM helix. In an effort to make slight improvements in the potency of the ligands, we also methylated and acetylated the N-terminal pyrrolidine amine of **2** to enable better occupation of a small hydrophobic pocket formed by Y308, C324, and W364 on the surface of EED (SI Fig. S3,S4 (UNC6008) and **5** (UNC6009), respectively)^10,16,31^. Additionally, three derivatives of UNC5114 were generated by introducing lysine side chain mimetics of varying lengths on the nitrogen atom of the N-terminal octahydroindole (Fig. 2 and SI Fig. S3).

### *In vitro* characterization of PRC2-EED-I363M activators

We prepared the trimeric wildtype PRC2 and PRC2-EED-I363M mutant to evaluate the ability of our synthesized ligands to perturb the catalytic activity of these complexes. In order to compare the activity of WT and mutant PRC2 we determined the k_cat_ values (turnover numbers) of both PRC2 complexes using a methyltransferase scintillation proximity assay as described in methods^32^. Not surprisingly, PRC2-EED-I363M showed significantly reduced catalytic activity relative to wildtype PRC2, with a k_cat_ value about 4-fold less than that of wildtype PRC2 (k_cat_ = 6 ± 1 h^−1^ and k_cat_ = 23 ± 1 h^−1^, respectively; Table 1; SI Fig. S4). We next investigated the ability of our synthetic ligands to influence the catalytic activity of the PRC2-WT or PRC2-EED-I363M complexes. Nine compounds along with methylated JARID2 were tested in total, including 7 compounds designed as PRC2 activators as well as the previously reported antagonists, UNC5114 and UNC4859. As expected, UNC5114 and UNC4859, antagonized the catalytic activity of PRC2-WT but showed minimal effects on PRC2-EED-I363M (SI Fig. S5).

**Table 1:** Determination of k_cat_ values in the presence of the active compounds using peptide or nucleosome as substrate.

Compound	k_cat_ (h^−1^)			
	H3(21–44)		Human nucleosome	
	PRC2-WT	PRC2-EED-I363M	PRC2-WT	PRC2-EED-I363M
DMSO (control)	24 ± 1	9 ± 1	2.5 ± 0.06	0.7 ± 0.04
**1** (UNC5635)	25 ± 0.5	20 ± 1	1.7 ± 0.05	1.3 ± 0.01
**2** (UNC5636)	24 ± 0.5	21 ± 1	1.6 ± 0.04	1.5 ± 0.02
**4** (UNC6008)	20 ± 1	12 ± 0.5	1.1 ± 0.04	0.8 ± 0.02

Data shown are mean ± SD, n = 3.

Among the 7 putative activators, two of the UNC4859 derivatives, **1** and **2**, resulted in a significant increase in PRC2-EED-I363M catalytic activity in a dose responsive fashion. Encouragingly, compared to methylated JARID2, these compounds not only selectively activated PRC2-EED-I363M and showed minimal effects on the activity of PRC2-WT, but also activated the mutant complex at a lower concentration (Fig. 2 and SI Fig. S5). The addition of a methyl group to the N-terminal proline of **2** (to give **4**, SI Fig. S3) resulted in weaker activation of PRC2-EED-I363M indicating that this modification is not well tolerated. In contrast, **5** in which the N-terminal proline of **2** is acetylated more closely resembles a weak inhibitor of PRC2-WT activity than an activator. Together, these results demonstrate that the simple introduction of a charged residue N-terminal to Kme3 in UNC4859 has the capability to convert UNC4859 from a PRC2 inhibitor to a mutant selective PRC2 activator; however, this modification alone is not sufficient for activation. Additionally, it seems likely that the basicity and/or charge of the proline amine contributes to the activation potential of these ligands, as acetylation at this position limits activation, potentially through reduced interactions with Y308 and C324 in EED.

Unlike the UNC4859 derivatives, the UNC5114 analogs were unable to activate PRC2-EED-I363M and inhibited PRC2-WT catalytic activity (Fig. 2 and SI Fig. S5). The octahydroindole ring in these compounds is quite constrained and the amine installed on this capping group is perhaps not well positioned to interact favorably with D136 and/or D140 on the EZH2 loop. Interestingly, as the length of the side chain becomes longer, the compounds show better inhibition of PRC2-WT. We speculated that an acidic patch formed by D362 on the surface of EED opposite to the aromatic cage forms a salt bridge with the positively charged lysine side-chain mimetics. This interaction appears to be more stable with the longer linker, which could be the reason why compound **3** showed the highest inhibition of PRC2-WT among these three compounds. We elaborate on this hypothesis further in the simulation section of this manuscript.

**Figure 3:**
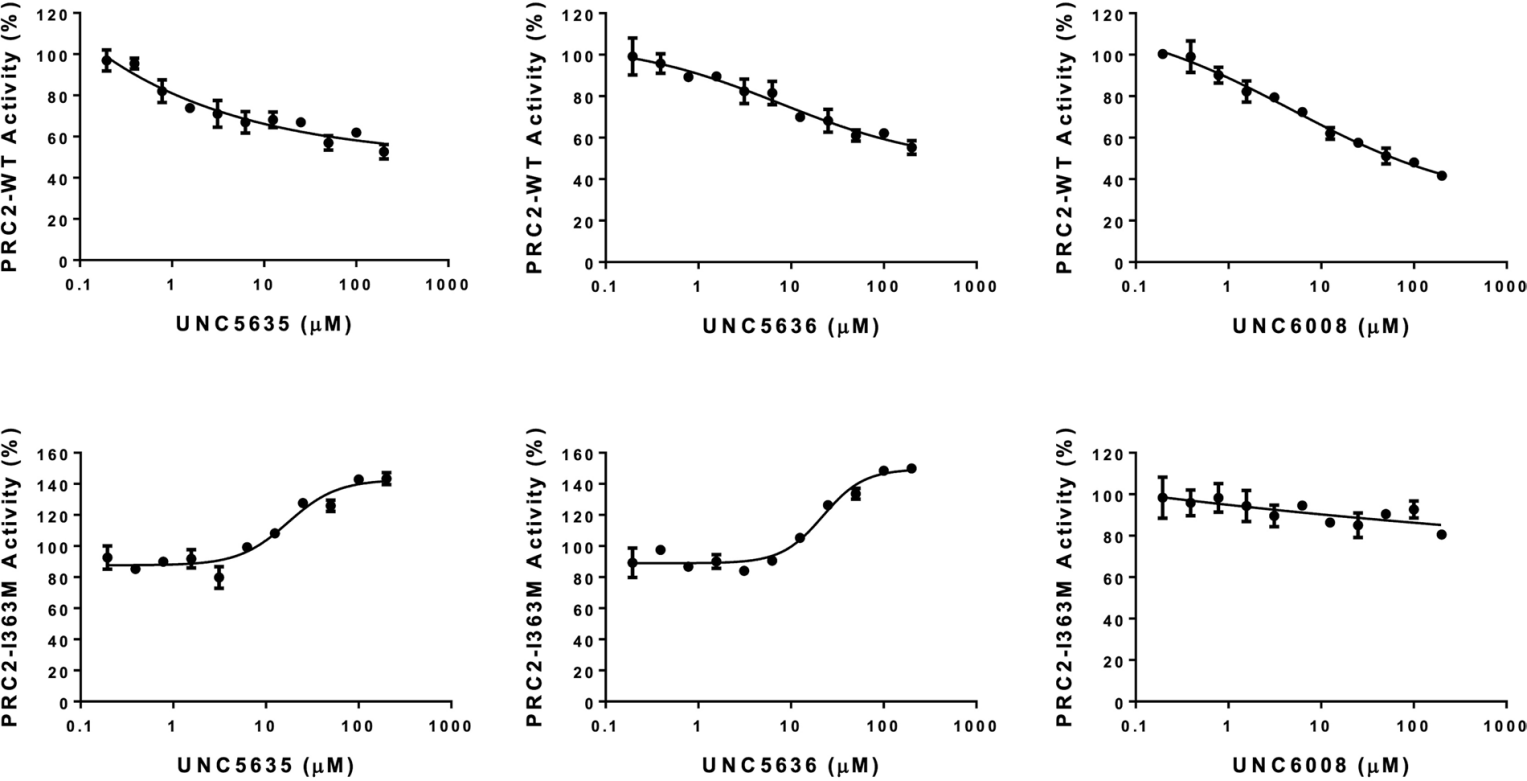
Effect of compound **1**, **2** and **4** on activity of PRC2-WT and PRC2-EED-I363M. Effect of the selected compounds on activity of PRC2-WT and PRC2-EED-I363M mutant were assessed using human nucleosome as a substrate. Experiments were performed in triplicate using 200 nM of enzyme, 2 µM 3H-SAM and 5 µM human nucleosome and 200 µM compounds incubated for 30 min at 23 °C in 20 mM Tris-HCl pH 8.0, 5 mM DTT, 0.01% Triton X-100.

We next determined EC_50_ values for the three UNC4859-derivatives compounds (**1**, **2**, and **4**) which showed promising PRC2-EED-I363M activation in order to better quantify how effectively these compounds activate PRC2-EED-I363M. Consistent with the catalytic activity assay results, **1** and **2** have similar EC_50_ values of 2.3 ± 0.4 µM and 2.4 ± 0.4 µM, respectively, while the EC_50_ value for **4** was 13 ± 2 µM (SI Fig. S6). To further explore these compounds as PRC2 activators, k_cat_ values were determined for wildtype and mutant PRC2 in the presence of 20 µM of each compound using peptide as substrate (SI Fig. S7A). While the compounds had minimal effect on the k_cat_ values for PRC2-WT complex, the PRC2-EED-I363M k_cat_ values increased by more than 2-fold in the presence of **1** and **2**, confirming their role as PRC2-EED-I363M catalytic activators (Table 1 and SI Fig. S7A). Similarly, we then determined the k_cat_ values using human nucleosomes as a substrate. In the presence of **1** and **2**, these k_cat_ values increased by about 2-fold whereas **4** showed no effect. In contrast, all three ligands negatively affected PRC2-WT catalytic activity (Table 1, Fig. 3 and SI Fig. S7B).

Next, to determine if the activation potential of these compounds for PRC2-WT versus PRC2-EED-I363M is based on their relative ability to bind the respective complexes, a fluorescein labelled analog of **2** was synthesized, **8** (UNC6419), and tested for PRC2-WT and PRC2-EED-I363M binding using a fluorescence polarization (FP) assay (SI Fig. S8A). The fluorescein was appended to a linker on the C-terminus of **2** via click chemistry, as we did not anticipate it would interfere with ligand binding at this position. Binding to PRC2 was determined in the presence or absence of SAM, SAH, or H3 unmodified peptide. In each case, **8** potently bound PRC2-WT with dissociation constants ranging from 156 nM to 226 nM depending on the experimental conditions (SI Fig. S8B). In contrast, no binding was detected for PRC2-EED-I363M. Since the top concentration of PRC2-EED-I363M achievable in the assay was 2 µM due to limitations in the solubility of recombinant EED-I363M, it is likely that this assay window is not sufficient to measure binding to PRC2-EED-I363M, as ligand binding is expected to be much weaker for the mutant due to the fact that I363M has been shown to significantly impair binding to H3K27me3^20^. Nevertheless, these ligands show activation of PRC2-EED-I363M to near wildtype levels.

### Computational simulations of ligand-bound PRC2-WT and PRC2-EED-I363M complexes

**Figure 4:**
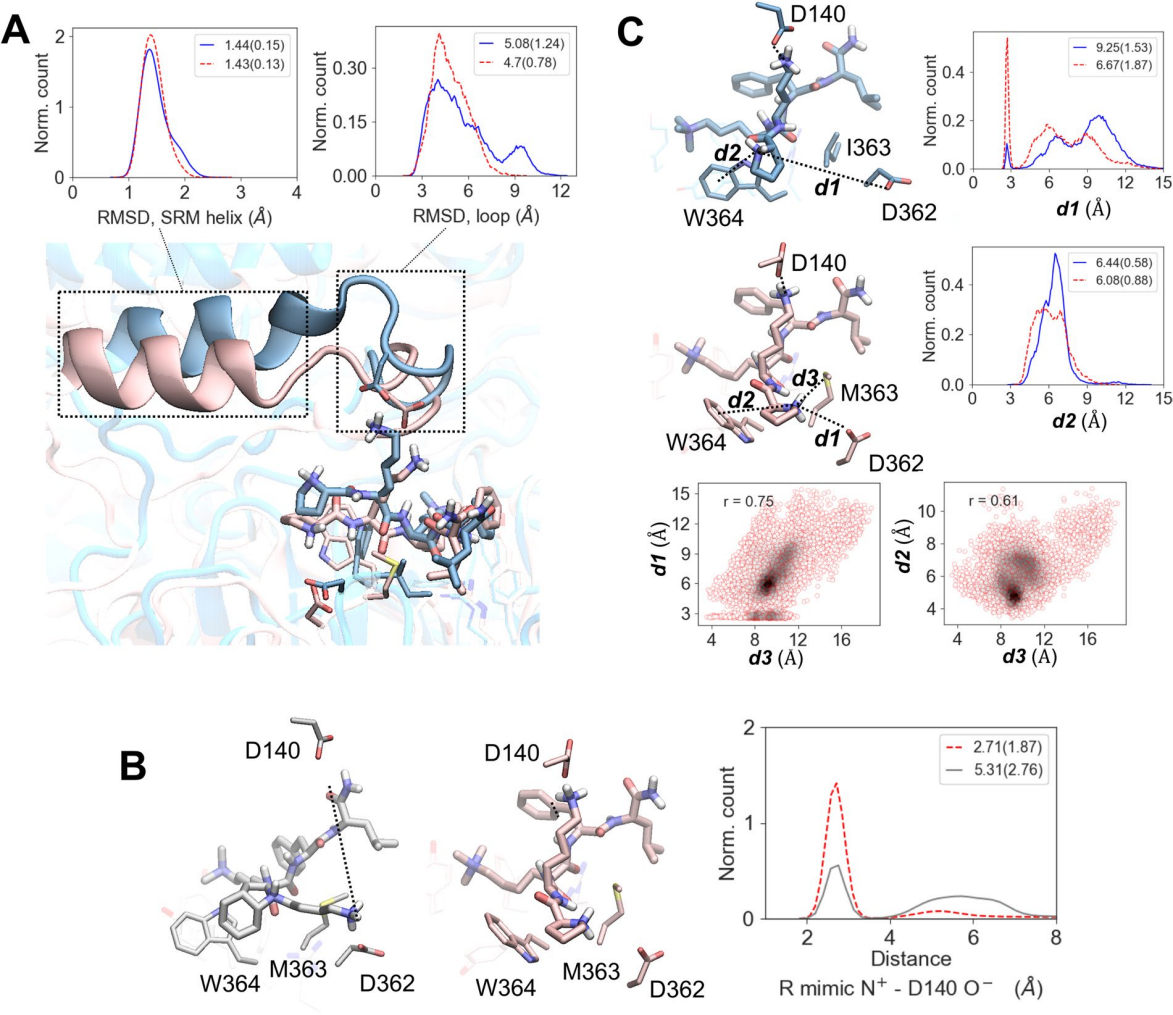
Insights from MD simulations. MD simulations indicate that compound **2** (UNC5636) is a selective activator of PRC2-EED-I363M mutant. **(A**) In contrast to mutant PRC2 (rose), the ligand in wildtype PRC2 (blue) causes a higher degree of conformational change in 136–142 loop of EZH2 leading to a partially unfolded SRM helix (RMSDs are with respect to the crystal structure of the active protein complex). In panels A and B wild type is represented with solid blue line and mutant—red dashed line. **(B)** Comparison of **2** with **3** in the context of mutant. In comparison with the activator molecule, **2** (rose), the arginine mimic in the inhibitor molecule, **3** (gray), has a lower propensity to form the key salt bridge responsible for the activation of the complex. **(C)** Protonated N-terminus of **2** forms more stable interactions with EED protein: a salt bridge with D362 (d1) and a cation-π interaction with W364 (d2). Both of these interactions seem to be induced (r correlation coefficient 0.75 and 0.61, respectively) by an electrostatic bond between the partially negatively charged sulfur atom of M363 and the positive pyrrolidine (d3), as well as by the shifted backbone of residues 362–364 of EED in the mutant (see text for details). The 2D correlation plots were constructed using kernel-density estimation with Gaussian kernels. In all panels the values in the legends of the distribution plots represent medians (and median absolute deviation in brackets). The molecular graphics were rendered with VMD 1.9.3 (http://www.ks.uiuc.edu/Research/vmd/)^42^.

We then sought to provide a structural rationale for the experimental results above and in particular to address the following questions: What is the structural basis for compound **2** (UNC5636) activating the mutant but not the wild type PRC2 complex? And, why is a similar compound **3** (UNC6083) an inhibitor, and not an activator, of both the mutant and wild type complexes? To address these questions, we performed molecular dynamics simulations of four molecular complexes, involving **2** and **3**, each in complex with both PRC2-WT and PRC2-EED-I363M, followed by comparative analyses of the respective MD trajectories. The key finding of our computational simulations is that indeed the dynamics of ligand **2** in complex with PRC2-EED-I363M differs significantly from both the dynamics of **2** in complex with WT PRC2 and that of **3** with the mutant PRC2. In particular, the most distinctive feature of the dynamic behavior of compound **2** in PRC2-EED-I363M is the persistent ionic interactions between the unmodified lysine side chain of **2** and D140 located in a flexible loop of EZH2 (residues 136–142), which drastically reduces the mobility of the loop, thus preserving the integrity of the adjacent SRM helix (Fig. 4A, SI Figs S9–11, and SI Movies S1, S2). In WT PRC2, **2** is still able to form similar interactions with the EZH2 loop. However, these contacts are significantly less frequent than in the mutant complex, thereby preventing effective stabilization of the SRM helix (in a large fraction of the simulation time the SRM helix was in a partially unfolded state (Fig. 4A)), which has been previously shown to be a key component of the EZH2 catalytically active state^15^. In contrast with **2**, **3** has an overall lower propensity for ionic interactions with the 136–142 loop of EZH2 (Fig. 4B and SI Fig. S11). Consequently, a typical binding pose of **3** favors ionic interactions of its lysine mimic with D362 of EED, thus leaving the 136–142 loop of EZH2 “unattended”, leading to only a partially folded state of the SRM helix (SI Figs S12, S13).

Taken together, our MD simulations provide a structural interpretation for the experimental data. First, compound **2** owes its activator potency in PRC2-EED-I363M to its ability to stabilize the SRM helix of EZH2, a key structural component of the catalytically active state. Due to a slightly different binding mode with EED, **3** does not induce SRM helix stabilization and, hence, does not show any activator effect on mutant PRC2. At the same time, since **3** competes for the same EED binding site with H3 and Jarid2, the endogenous PRC2 activators, it shows a dose-dependent inhibitory potency on the WT enzyme. Finally, the system involving compound **2** bound to PRC2-WT represents an intermediary case where the synthetic ligand can only intermittently stabilize the SRM helix, so that its activator potency does not exceed that of the endogenous peptide activators. Hence, **2** is ‘invisible’ in the assay with the H3 peptide as the substrate. Additionally, the endogenous peptide is probably a better activator when whole nucleosomes are used as the substrate, thus making **2** appear as a weak inhibitor in this assay (Table 1 and Fig. 3).

We also sought to understand how the EED-I363M mutation reduces the sensitivity of PRC2 to its endogenous activators but favors the activator potency of **2**. A closer look at the EED mutation site shows that the mutant’s methionine side chain (that occupies a significantly larger dynamic volume than the wild type isoleucine) significantly increases the mobility of the backbone atoms of the EED residues 362–364. This higher mobility enabled the side chains of D362 and W364 to move closer to the positively charged pyrrolidine group of **2** and form respectively a salt bridge and a cation-π interaction (***d1*** and ***d2***, respectively, in Fig. 4C and SI Figs S15, S16). This same increased mobility of the EED residues 362–364 appears to destabilize the endogenous activator’s binding to EED leading to an overall decrease in PRC2 activity.

## Discussion

We made use of structure-based design and computational simulations to develop small-molecule ligands able to selectively activate PRC2 carrying an oncogenic LOF mutation of its EED subunit. Designed compounds were synthesized and biochemically characterized to confirm their efficacy and selectivity, and the use of structural analysis of PRC2 complexes as well as molecular dynamics simulation with the most activating ligand, compound **2**, and the non-activating compound, compound **3**, allowed us to propose molecular details underpinning selectivity and allosteric activation capability. The phenotypic outcome of EED-I363M in humans is hematologic malignancies caused by impaired propagation of repressive histone marks^30^, hence the discovery of allosteric activators for EED-I363M provides an approach to reverse the phenotype of this mutant. However, the tool compounds we describe here are peptidic and contain positive charges including a quaternary amine that are unsuited for membrane permeability, consequently limiting their application in cellular studies^33^. Thus, further improvements in drug-like properties that permit cellular penetration are necessary to test whether allosteric activation of mutant PRC2 can be achieved in cells and whether this wouldindeedreverse the mutant phenotype. Nevertheless, encouragingly, we have proven that the introduction of positively charged side chain at the –1 position in the previously reported PRC2 allosteric antagonist, UNC4859, has converted the molecule into a mutant specific allosteric agonist. We anticipate that these tool compounds and proof-of-concept for activation of EED-mutant LOF PRC2 will inspire the development of more drug-like EED-I363M activators in an effort to restore PRC2 function in disease relevant settings, such as MDS^20,30^.

## Methods

### Protein expression and purification

DNA fragments encoding the genes of the full lengths of two components of the PRC2 complex, EED(1–441) and EZH2(1–751) were cloned into pFastBac-Dual (Invitrogen) and the full length of N-flag SUZ12(1–739) were cloned into pFastBac HT A (Invitrogen). The resulting plasmids were transformed into DH10Bac™ Competent *E. coli* (Invitrogen) and recombinant viral DNA bacmids were purified and followed by a recombinant baculovirus generation in Sf9 insect cells. Sf9 cells grown in HyQ® SFX insect serum-free medium (ThermoScientific) were co-infected with 10 ml of each P3 viral stocks per 0.8 L of suspension cell culture and incubated at 27 °C using a platform shaker set at 100 RPM. The cells were collected after 72 hours of post-infection time when viability dropped to 70–80%. Harvested cells were re-suspended in PBS, 1X in-house protease inhibitor cocktail (100 X protease inhibitor stock in 70% ethanol contains 0.25 mg/ml Aprotinin, 0.25 mg/ml Leupeptin, 0.25 mg/ml Pepstatin A and 0.25 mg/ml E-64) and 2X Roche complete EDTA-free protease inhibitor cocktail tablet. The cells were lysed chemically by rotating for 30 minutes with NP-40 (final concentration of 0.6%) and 50 U/mL Benzonase nuclease (Sigma), 2 mM 2-mercaptoethanol and 10% Glycerol followed by sonication at frequency of 7 (10 seconds on/10 seconds off) for 2 minutes (Sonicator 3000, Misoni). The crude extract was clarified by high-speed centrifugation (60 min at 36,000 × g at 4 °C) by Beckman Coulter centrifuge. The recombinant protein was purified by incubating the cleared lysate with equilibrated Anti-FLAG M2 Affinity agarose gel (Sigma, Cat # A2220) for 3 hours rotating, followed by washing with 10 column volumes of TBS (50 mM Tris-HCl, with 150 mM NaCl, pH 7.4) with 2 mM 2-mercaptoethanol, 1X in-house protease inhibitor cocktail and 1X Roche complete EDTA-free protease inhibitor cocktail tablet. The recombinant protein was eluted by competitive elution with a solution containing 100 µg/ml FLAG peptide (Sigma, Catalog # F4799) in 20 mM Tris pH 7.4, 150 mM NaCl, 5% glycerol. 4 mM DTT was added to eluent in the end. Protein purity was judged by SDS–PAGE. The protein complex was then concentrated, flash frozen and stored at −80 °C. The same protocol was used to express and purify PRC2-EED-I363M mutant.

### Catalytic assay

Methyltransferase activity assay for WT PRC2 (EED/EZH2/SUZ12) or mutant PRC2-EED-I363M (EED/EZH2/SUZ12) was performed by monitoring the incorporation of tritium-labeled methyl group to lysine 27 of H3 (21–44) peptide using Scintillation Proximity Assay (SPA) or to human nucleosome using a filter-based assay. The enzymatic reactions were performed at 23 °C with 1 hour incubation of 10 µl reaction mixture in 20 mM Tris-HCl, pH 8, 5 mM DTT, and 0.01% Triton X-100 containing 2 µM of ^3^H-SAM (Cat.# NET155V250UC; Perkin Elmer; www.perkinelmer.com), 1 µM of biotinylated H3 (21–44), 20 nM PRC2-WT or PRC2-mutant. To stop the reactions, 10 µL of 7.5 M Guanidine hydrochloride was added, followed by 200 µl of buffer (20 mM Tris, pH 8.0), mixed and transferred to a 96-well Streptavidin coated Flash-plate (Cat. # SMP410A001PK, PerkinElmer, http://www.perkinelmer.ca). After mixing, the mixtures in Flash-plate were incubated for 2 hours and the CPM counts were measured using Topcount plate reader (Perkin Elmer, www.perkinelmer.com).

A filter-based assay was used for human nucleosome as a substrate. The reactions were carried out at 23 °C with 0.5 hour incubation of 10 µl reaction mixture in 20 mM Tris-HCl, pH 8, 5 mM DTT, and 0.01% Triton X-100 containing 2 µM of ^3^H-SAM (Cat.# NET155V250UC; Perkin Elmer; www.perkinelmer.com), 5 µM of human nucleosome, 200 nM PRC2-WT or PRC2 mutant. To stop the reactions, 50 µl of 10% TCA was added, mixed and transferred to filter-plates (Millipore; Cat.# MSFBN6B10; www.millipore.com). Plates were centrifuged at 2000 rpm (Allegra X-15R - Beckman Coulter, Inc.) for 2 min followed by two additional 10% TCA wash and one ethanol wash (180 µl) followed by centrifugation. Plates were dried and 100 µl MicroO (MicroScint-O; Cat.# 6013611, Perkin Elmer; www.perkinelmer.com) was added to each well, centrifuged and removed. 70 µl of MicroO was added again and CPM was measured using Topcount plate reader.

The CPM counts in the absence of compound for each dataset were defined as 100% activity. In the absence of the enzyme, the CPM counts in each data set were defined as background (0%). All enzymatic reactions were performed in triplicate and the data were analyzed by GraphPad Prism 7 software.

### Binding Assay

The binding experiments were performed in a total volume of 10 μl binding buffer (20 mM Tris pH 8.0 supplemented with 0.01% Triton X-100 and 5 mM DTT) in 384-well black polypropylene PCR plates (Axygen, Cat. PCR-384-BK). The binding of FAM-labelled ligand, **8** (UNC6419) to WT- and EED-I363M mutant trimeric PRC2 complexes was evaluated by monitoring the fluorescence polarization (FP) signal in the presence of 40 nM FL-UNC6419. FP was performed using a Biotek Synergy 4 after 30 minutes incubation at ambient temperature using excitation and emission wavelengths of 485 nm and 528 nm, respectively.

### Molecular dynamics simulation

All molecular dynamics (MD) simulations were performed in Gromacs 2018.2 simulation package using CHARMM22 protein force field^34^. The volta GPU nodes on UNC Longleaf supercomputer cluster were utilized. Each simulation was performed on a combination of 1 GPU and 8 associated CPUs, which provided the optimal recourses-speed correlation. The crystal structure of human PRC2 complex (PDB ID 5HYN)^10^ was used as the starting structures in the simulations. Maestro Schrödinger software (release 2016-2, Schrödinger, LLC: New York, NY) was used for homology modeling of some of the missing loops in EZH2 and to perform I363M mutation in EED. The ligands were designed using Build function in Maestro starting from the structure of UNC4859 bound to EED (PDB ID 5TTW) upon alignment. The modified histone tail parameters compatible with CHARMM ff were obtained from Dejaegere *et al*.^35^ and adapted for use in Gromacs. These parameters were necessary in order to correctly represent K116me3 of Jarid2 in our simulations. MMFF parameters for the ligands and SAH molecule compatible with CHARMM22 were generated using Swissparam^36^. End caps were added to both termini of each protein. The protein complex was minimized in vacuum using steepest decent algorithm for 5,000 steps or until the maximum force of 1,000 kJ*mol^−1^*nm^−1^ was reached. The molecular systems were then solvated in TIP3P water, counterions were added for system neutrality, and NaCl was added by replacing water molecules in order to mimic 0.15 M physiological conditions. The total system size became ~230,000 atoms. Solvent minimization was then performed, followed by a two-step equilibration, during which all heavy atoms of the system, excluding those of water and counterions, were restrained: 0.1 ns in NVT ensemble using the modified Berendsen thermostat^37^ set at constant 310 K, and 1 ns in NPT ensemble at constant 1 atm and 310 K using the Parinello-Rahman pressure coupling^38^. All simulations were conducted using the Leapfrog integrator in periodic boundary conditions. The 12-6 Lennard-Jones potential was used to describe the vdW interactions, and the nonbonded cutoff distance was set at 0.1 nm. The particle mesh Ewald algorithm^39^ controlled the long-range electrostatic interactions. Bonds involving hydrogen atoms were constrained using the linear constraint solver algorithm (LINCS)^40^. The production simulations were conducted in NPT ensemble with all atoms free to move. Each of the six systems, each PRC2 wild type and mutant in complex with Jarid2, and each wild type and mutant with each of the active and inactive ligands (**2** (UNC5636) and **3** (UNC6083)), were subjected to five independent 500 ns long MD runs totaling 15 μs cumulative simulation time. For the analysis we removed the frames containing unbound ligands (RMSD >0.8 nm). Gromacs’s trajectory analysis tools, MDTraj^41^ along with *in-house* bash and python scripts were used for data analysis and matplotlib for plotting. Molecular visualization and generation of graphics and videos were performed in VMD 1.9.3 (http://www.ks.uiuc.edu/Research/vmd/)^42^.

### Solid-phase peptide synthesis

Peptidomimetic synthesis was conducted on Fmoc Rink amide resin on polystyrene beads (100 mg per peptide, Anaspec). The resin was rinsed once with dichloromethane (DCM), drained, and swollen in DCM for 5 min followed by equilibration in N,N-dimethylformamide (DMF) for another 5 min. Fmoc protecting group on Fmoc Rink amide resin was first removed in a solution of 2.5% 1,8-diazabicycloundec-7-ene (DBU) and 2.5% pyrrolidine in 8 mL of DMF for 10 min. Then, the resin was filtered and washed twice with DMF, methanol, DMF, and DCM (8 mL each) before adding the first amino acid for coupling. Fmoc-protected amino acids (3 eq) except Fmoc-Arg(Pmc)-OH were preactivated with HBTU (3 eq), HOAt (3 eq), and DIPEA (10 eq) for 5 minutes with periodic swirling in 5 mL of DMF and 3 mL of DCM. For Fmoc-Arg(Pmc)-OH coupling, we avoided this longer pre-incubation to prevent formation of the intramolecular cyclization by-product. The solution was then added to the resin and left on a shaker at room temperature for 1 hr. The resin was filtered and washed twice with DCM, DMF, methanol, and DMF again (8 ml each). Fmoc protecting groups on N-terminal amino acid were removed in a solution of 2.5% 1,8-diazabicycloundec-7-ene (DBU) and 2.5% pyrrolidine in 8 mL of DMF for 10 min. Then, the resin was filtered and washed twice with DMF, methanol, DMF, and DCM (8 mL each) before adding the next amino acid for coupling. Following installation of the capping residue, the resin was rinsed 10 times with DCM. Cleavage cocktail (95% trifluoroacetic acid, 2.5% triisopropylsilane, and 2.5% water) was added to the resin, the mixture was left on the shaker for 2 hours, and the filtrate was collected. Resin was rinsed twice with DCM and filtrates were pooled then concentrated under vacuum. The mixture was dissolved in water, washed with ether, and the peptidomimetics were extracted with water (3 × 10 mL). The aqueous layers were combined then concentrated *in vacuo*, and purified via preparative high performance liquid chromatography (10–100% acetonitrile in H_2_O + 0.1 trifluoroacetic acid). The solvent was removed *in vacuo* and dissolved in water, and lyophilized to yield the desired peptidomimetic products.

## Supplementary information

Supplementary Information

Video S1

Video S2
