# Supplementary material for: Discovery of selective activators of PRC2 mutant EED-I363M
Source: Sci Rep. 2019 Apr 25;9:6524. doi: 10.1038/s41598-019-43005-z (PMC6484020; doi:10.1038/s41598-019-43005-z)
Supplement: Supplementary file 1 — Supplementary Information [file 41598_2019_43005_MOESM1_ESM.pdf]

Discovery of selective activators of PRC2 mutant EED-I363M

Junghyun L. Suh[†,1], Kimberly D. Barnash[†,1,2], Tigran M. Abramyan[1], Fengling Li[3], Juliana The[3], Isabelle A. Engelberg[1], Masoud Vedadi[3], Peter J. Brown[3], Dmitri B. Kireev[1], Cheryl H. Arrowsmith[3,4], Lindsey I. James[1], and Stephen V. Frye*[1]*

[1]Center for Integrative Chemical Biology and Drug Discovery, Division of Chemical Biology and Medicinal Chemistry, UNC Eshelman School of Pharmacy, University of North Carolina at Chapel Hill, Chapel Hill, North Carolina 27599, USA. [2]Current Contact Information: Foghorn Therapeutics, Cambridge, MA 02142, USA. [3]Structural Genomics Consortium, University of Toronto, Toronto, Ontario M5G 1L7, Canada. [4]Princess Margaret Cancer Centre and Department of Medical Biophysics, University of Toronto, Toronto, Ontario M5G 2M9, Canada. [†]These authors contributed equally to this work.

Table of Contents

Supplementary Figure S1. 3
Supplementary Figure S2. 4
Supplementary Figure S3. 5
Supplementary Figure S4. 5
Supplementary Figure S5. 6
Supplementary Figure S6. 7
Supplementary Figure S7. 8
Supplementary Figure S8. 9
Supplementary Figure S9. 10
Supplementary Figure S10. 10
Supplementary Figure S11. 10
Supplementary Figure S12. 11
Supplementary Figure S13. 11
Supplementary Figure S14. 12
Supplementary Figure S15. 12
Supplementary Figure S16. 12
General Chemistry Procedures 13
Analysis of Products 13
^{1}H NMR Spectra 18
Supplementary Movies 22
References 23

The following data were calculated from the molecular dynamics simulations:

RMSDs:

- EZH2 SRM helix (residues 143-153) upon SRM Cα alignment (**Fig. S2C**, **Fig. S9**)
- EZH2 SRM helix upon EED Cα alignment (**Fig. S1**, **Fig. S2D**, **Fig. S9**);
- EZH2 loop, residues 136-142, upon EED Cα alignment (**Fig. S10**);
- Ligand RMSD (**Fig. S14**);

Minimum distances (designated as Distance on figures):

- Jarid2 R115 N^+—EZH2 D136 O^- (**Fig. S2A**);
- Jarid2 R115 N^+—EZH2 D140 O^- (**Fig. S2B**);
- Ligand's R mimic N^+—EZH2 D140 O^- (**Fig. S11**);
- Ligand's R mimic N^+—EED D362 O^- (**Fig. S12**, **Fig. S13**);
- Ligand's N-terminus N^+—EED D362 O^- (**Fig. S15**);
- Ligand's N-terminus N^+—EED W364 benzene ring (**Fig. S16**);

The legends of the figures represent median values of the parameter (and median absolute deviation in the brackets) for each of the five independent simulation runs of each simulated system (PRC2 WT (WT) with Jarid2, PRC2 I363M (I363M) mutant with Jarid2, WT-**3** (UNC6083), I363M-**3**, WT-**2** (UNC5636), I363M-**2**). For clearer representation of the time series lines the Savitzky-Golay filter with window size 171 and polynomial order of 3 was used to remove high frequency noise from the data, and the full data is also plotted using transparent lines.

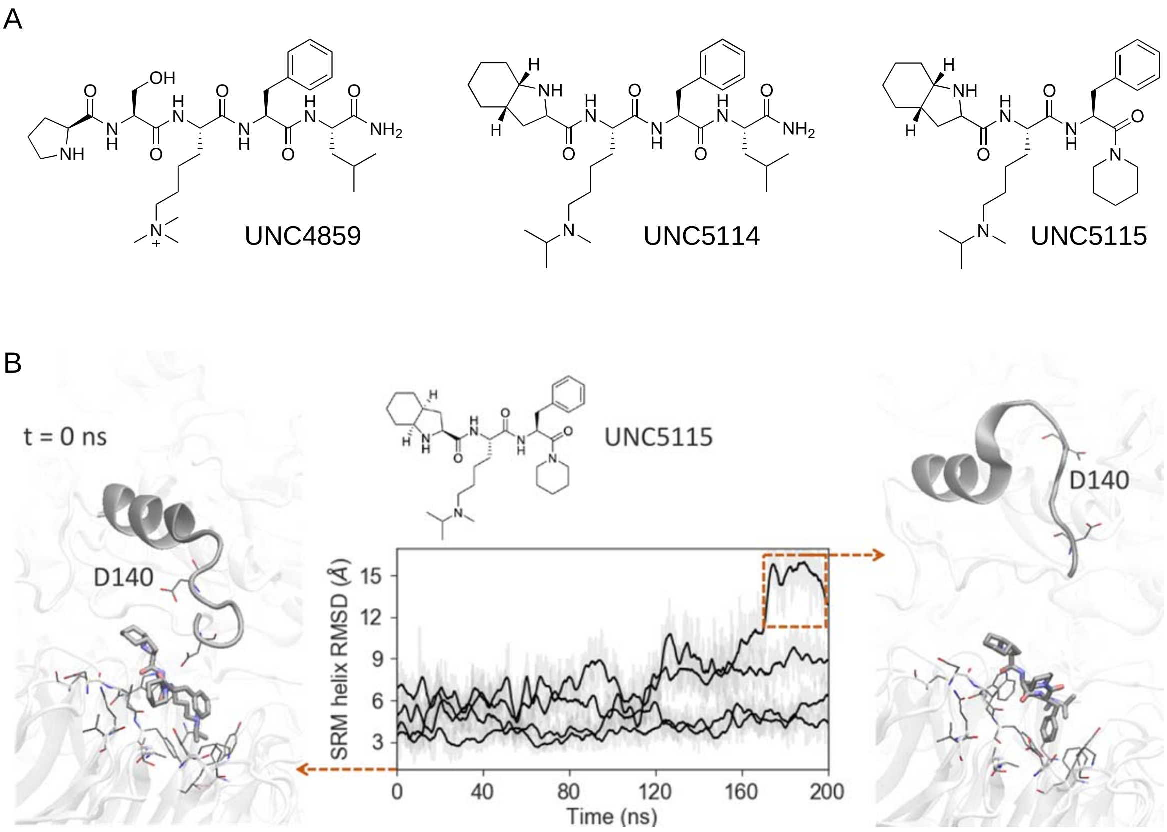

Supplementary Figure S1. (A) Previously reported EED allosteric inhibitors: UNC4859, UNC5114, and UNC5115 (1). **(B)** UNC5115 competes with Jarid2 in binding to EED leading to PRC2 inhibition (1). By lacking an arginine mimic, the ligand is unable to capture D140 which leads to SRM helix unfolding shown in our simulation studies (higher values of RMSD with respect to the crystal structure of Jarid2 bound active complex, PDB ID 5HYN (2)). The molecule possessing ˜1 μM EED binding affinity served as a starting structure for PRC2 activator design.

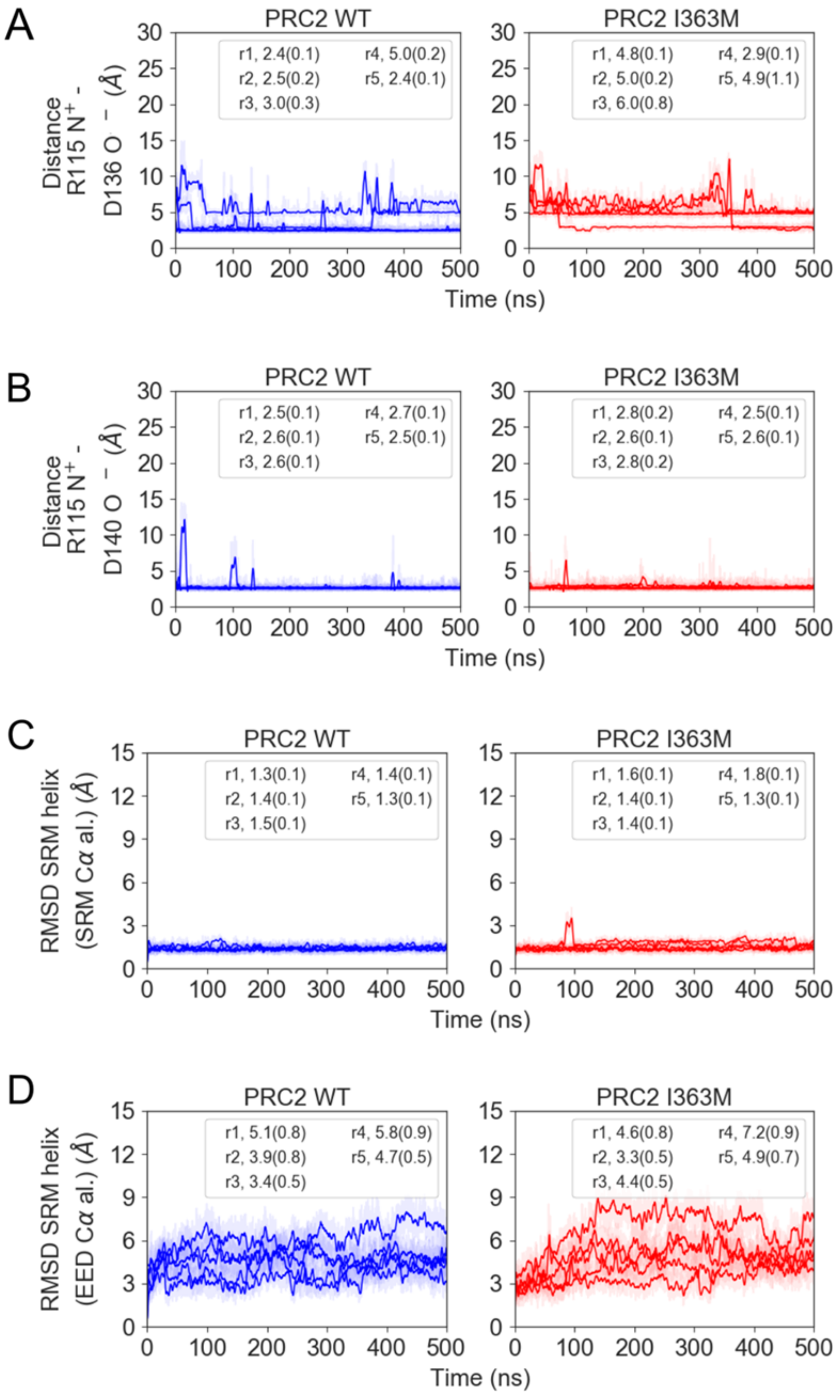

Supplementary Figure S2. Simulation results of PRC2 and PRC2 I363M systems with Jarid2 peptide. Distances between N^+ of Jarid2 peptide and O^- of both **(A)** D136 and **(B)** D140 residues of EZH2. RMSD of EZH2 SRM helix following both **(C)** EZH2 SRM helix Cα and **(D)** EED Cα alignments.

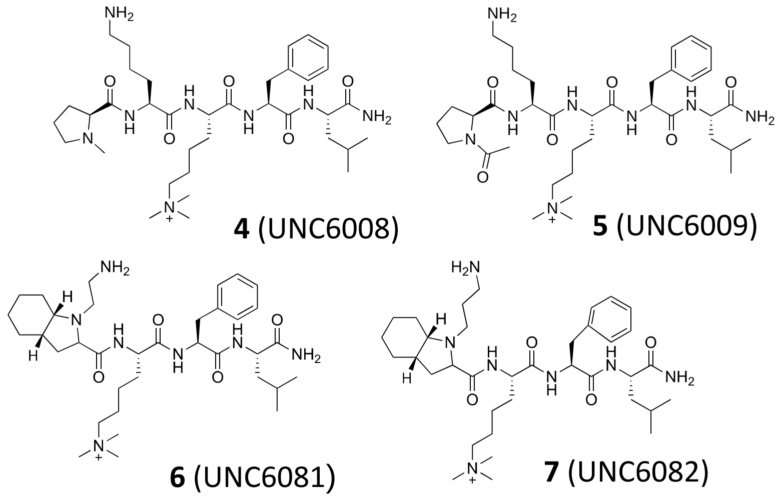

Supplementary Figure S3. Full structures of UNC4859-derivatives and UNC5114-derivatives

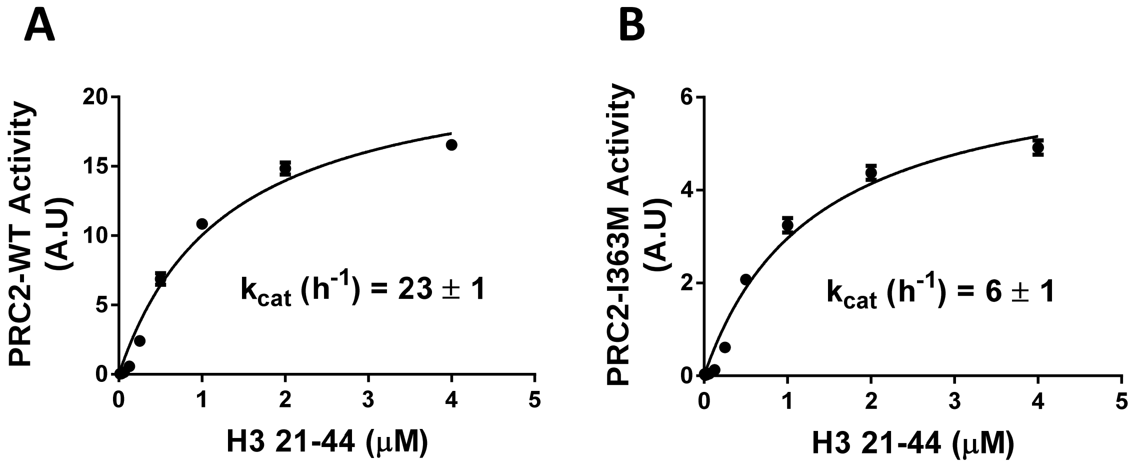

Supplementary Figure S4. Comparison of the wild-type (WT) and EED-I363M mutant PRC2 activities. We determined the kinetic parameters for **(A)** WT-PRC2 and **(B)** EED-I363M mutant PRC2 using H3 peptide residues 21 to 44 (H3 21-44) as substrate. WT-PRC2 was 3.8-fold more active (k_{cat} of 23 ± 1 h^{-1}) than EED-I363M mutant (k_{cat} of 6 ± 1 h^{-1}). Assays were performed in triplicate using trimeric (EED/EZH2/SUZ12) PRC2 complexes. Assay conditions: 20 nM enzyme, 5 μM ^{3}H-SAM and various concentrations of biotinylated-H3 (21-44) up to 4 μM in 20 mM Tris-HCl pH 8.0, 5 mM DTT, 0.01% Triton X-100). Data shown are mean ± SD.

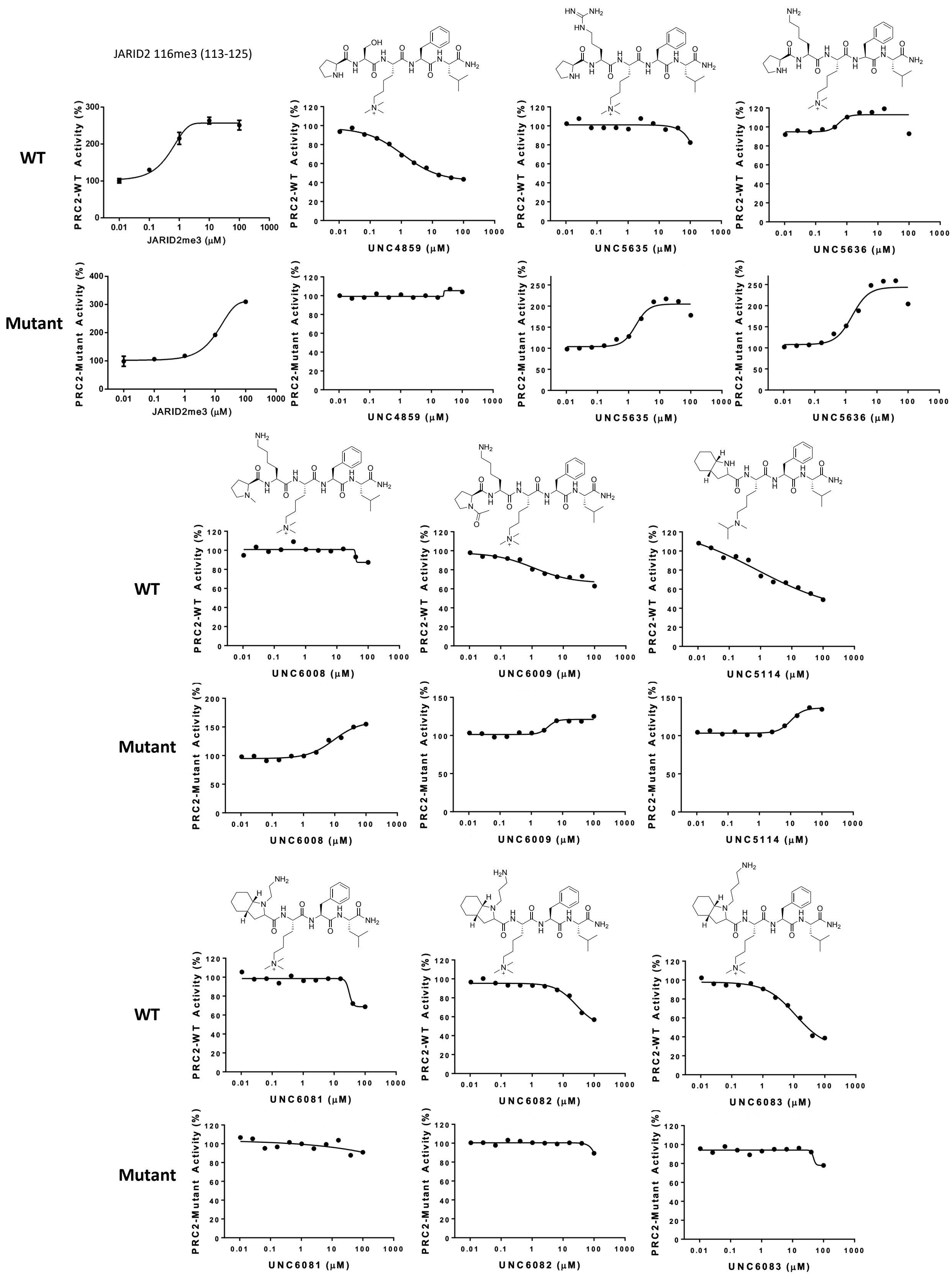
JARID2 116me3 (113-125)
WT
Mutant
PRC2-WT Activity (%)
PRC2-Mutant Activity (%)
JARID2me3 (μM)
UNC4859 (μM)
UNC5635 (μM)
UNC5636 (μM)
UNC6008 (μM)
UNC6009 (μM)
UNC5114 (μM)
UNC6081 (μM)
UNC6082 (μM)
UNC6083 (μM)

Supplementary Figure S5. Testing the effect of 9 compounds on activity of WT- and EED-I363M mutant PRC2 complexes. Activities of WT- and mutant-PRC2 in the presence of each compound are normalized to activity of each protein complex in the absence of compound as 100%. Experiments were performed using 20 nM of each WT or mutant PRC2 trimeric (EED/EZH2/SUZ12) complexes, 2 µM ^{3}H-SAM, 1 µM biotin-H3 (21-44), and up to 100 µM of compounds, 20 mM Tris-HCl pH 8.0, 5 mM DTT and 0.01% Triton X-100.

Compound	EC_{50} (µM)	Hill Slope	Top %	Bottom %
UNC5635	2.3 ± 0.4	1.7	222	100
UNC5636	2.4 ± 0.4	1.9	252	100
UNC6008	13 ± 2	0.8	197	100

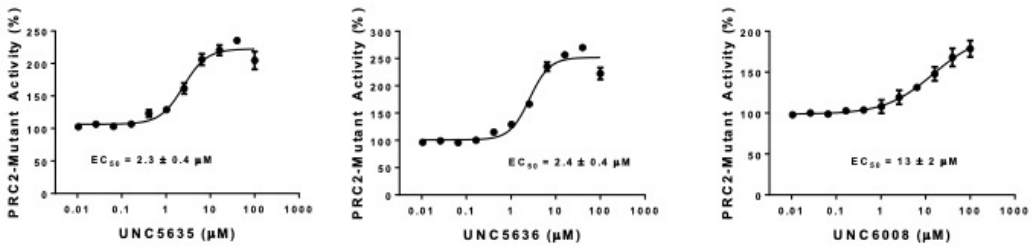

Supplementary Figure S6. EC_{50} determination for activators. The EC_{50} values were determined for **1** (UNC5635), **2** (UNC5636) and **4** (UNC6008). Assay conditions: 20 nM EED-I363M trimeric PRC2 mutant (EED/EZH2/SUZ12), 5 µM ^{3}H-SAM, 2 µM biotin-H3 (21-44) and compound concentrations up to 100 µM. Reactions were incubated for 30 min at 23°C in 20 mM Tris-HCl pH 8.0, 5 mM DTT, 0.01% Triton X-100). Experiments were performed in triplicate. Data shown are mean ± SD.

A. H3$_{21-44}$K27

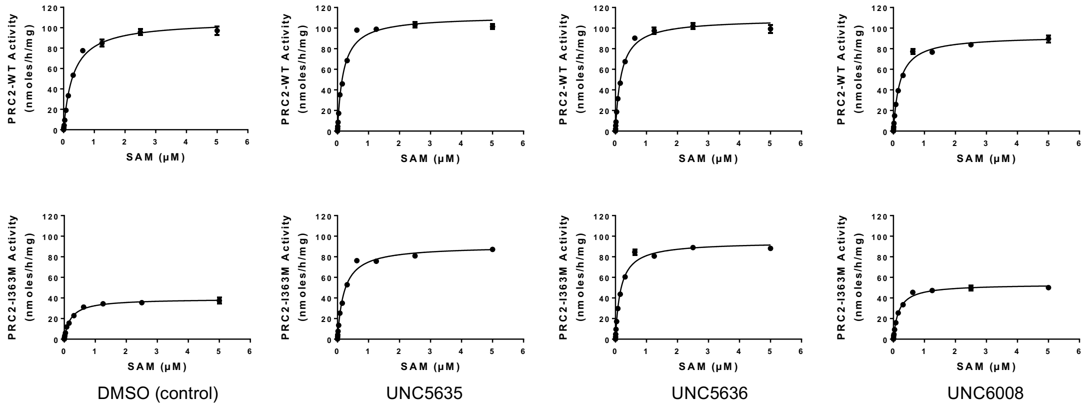

B. Human nucleosome

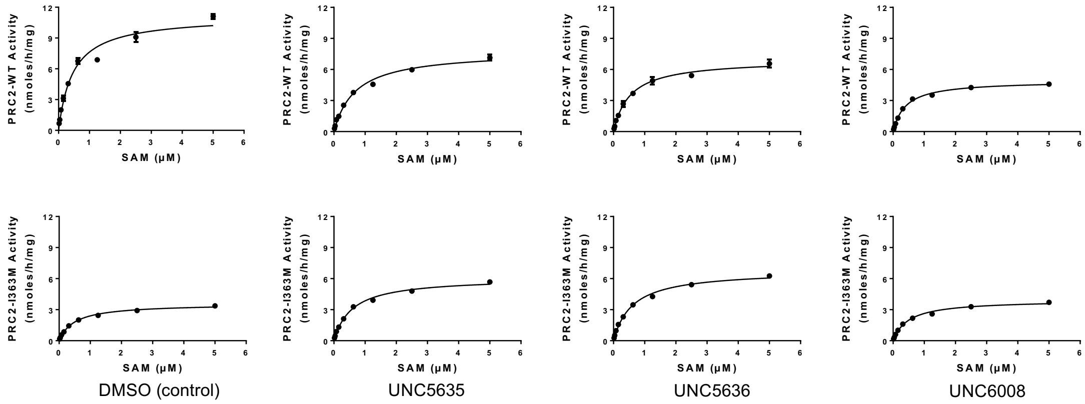

Supplementary Figure S7. Determination of k_{cat} values for WT- and EED- I363M mutant trimeric PRC2 complexes in the presence of activators. The k_{cat} values were determined using **(A)** H3 (21-44) in the presence of 20 µM of each compound or **(B)** human nucleosome as a substrate in the presence of 200 µM of each compound. All k_{cat} values are presented in **Table 1**. Experiments were performed in triplicate under the following assay conditions:

(A) Peptide as substrate: 20 nM wildtype or EED-I363M mutant PRC2 (EED/EZH2/SUZ12), 2 µM biotinylated-H3 (21-44) peptide, various concentrations of ^{3}H-SAM (up to 5 µM).

(B) Human nucleosome as a substrate: 200 nM wildtype or EED-I363M mutant trimeric PRC2 (EED/EZH2/SUZ12), various concentrations of ^{3}H-SAM (up to 5 µM) and 5 µM human nucleosome.

Reactions with both substrates were incubated for 30 min at 23°C in 20 mM Tris-HCl pH 8.0, 5 mM DTT, 0.01% Triton X-100.

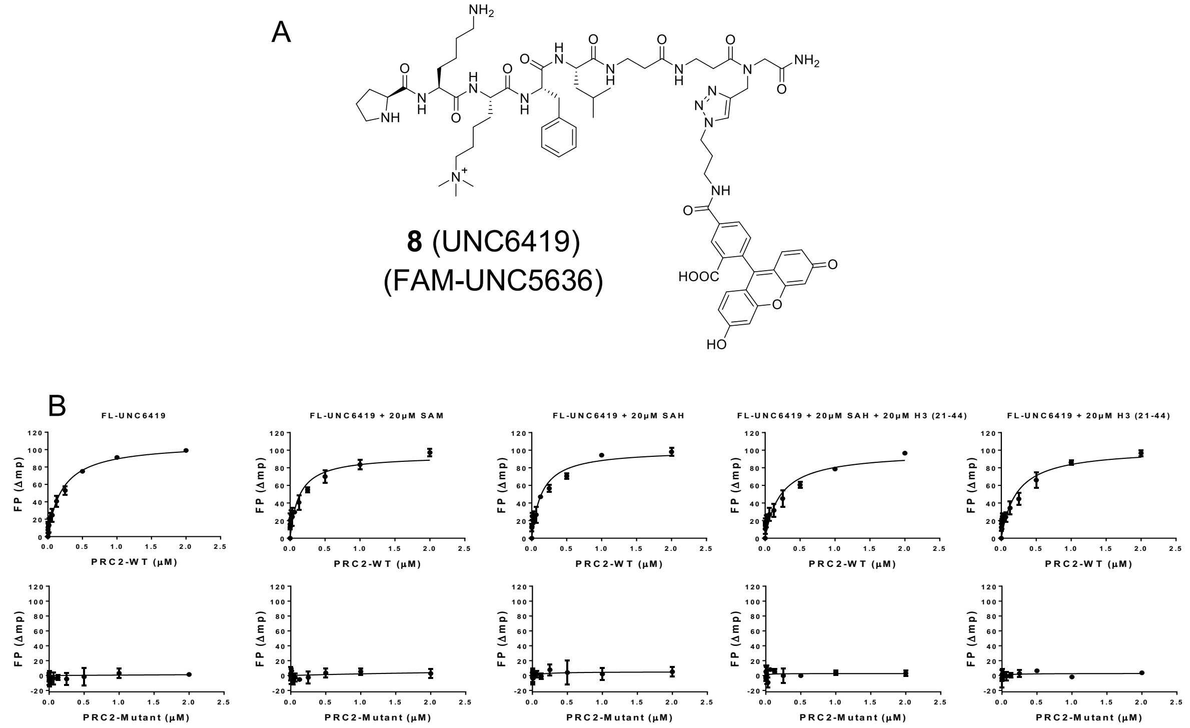

Ligand	PRC2-WT		PRC2-I363M	
	K_d (nM)	B_{max} (Δmp)	K_d (nM)	B_{max} (Δmp)
FL-UNC6419	**211 ± 64**	**108 ± 9**	NA	NA
FL-UNC6419 + 20μM SAM	**156 ± 38**	**95 ± 9**	NA	NA
FL-UNC6419 + 20μM SAH	**193 ± 48**	**101 ± 7**	NA	NA
FL-UNC6419 + 20μM SAH + 20μM H3 (21-44)	**226 ± 75**	**98 ± 8**	NA	NA
FL-UNC6419 + 20μM H3 (21-44)	**218 ± 17**	**102 ± 2**	NA	NA

Supplementary Figure S8. Evaluating binding of **8** (UNC6419) to WT- and EED-I363M mutant trimeric PRC2 complexes. Binding of **(A) 8**, a FAM-labelled ligand, to both WT and mutant trimeric PRC2 complexes were assessed by **(B)** monitoring the changes in fluorescence polarization signal under specified conditions. Increase in fluorescence polarization signal was observed when increasing concentrations of wild-type, but not mutant, PRC2 (EED/EZH2/SUZ12) was tested in the presence of 40 nM of **8**. All K_d values are presented in the above table. Assays were performed in triplicate in 20 mM Tris-HCl pH 8.0, 5 mM DTT, 0.01% Triton X-100. Data shown are mean ± SD.

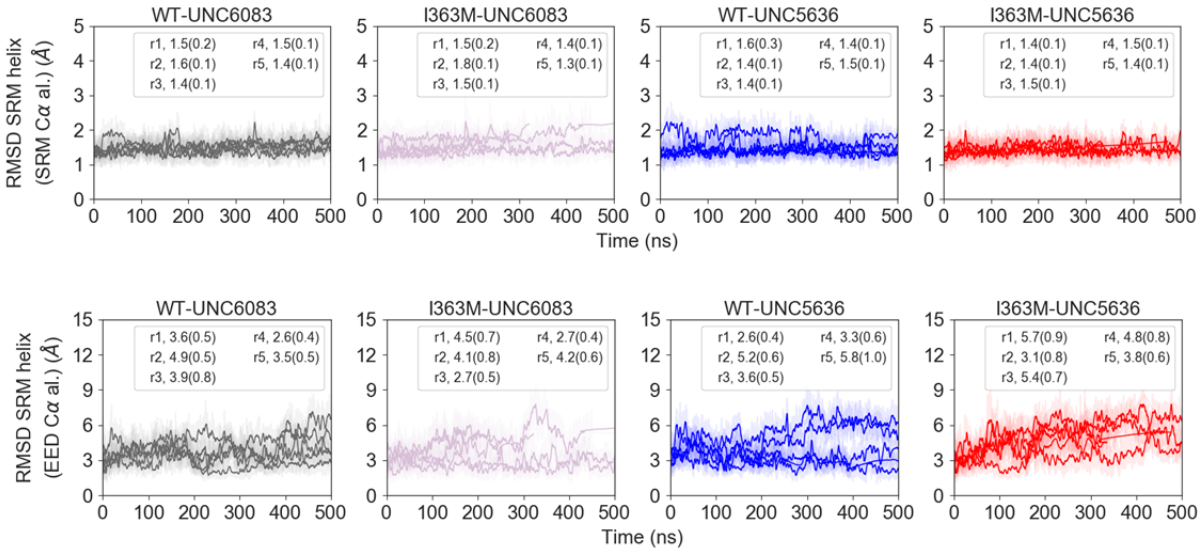

Supplementary Figure S9. RMSD of EZH2 SRM helix (both upon EZH2 SRM helix Cα (top plots) and EED Cα alignment (bottom plots)).

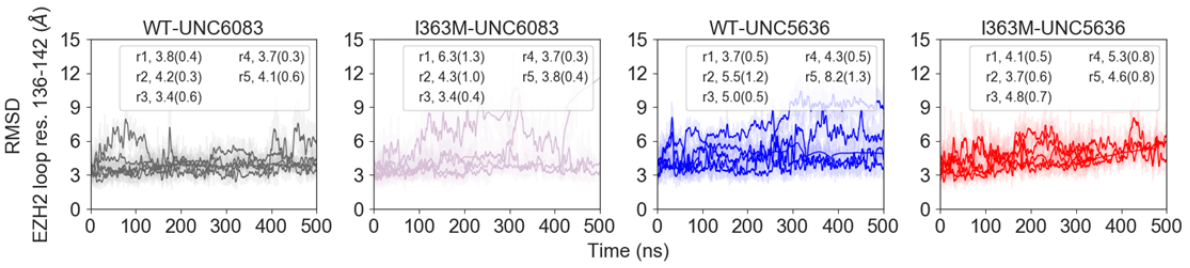

Supplementary Figure S10. RMSD of EZH2 loop, residues 136-142, leading to SRM helix (upon EED Cα alignment).

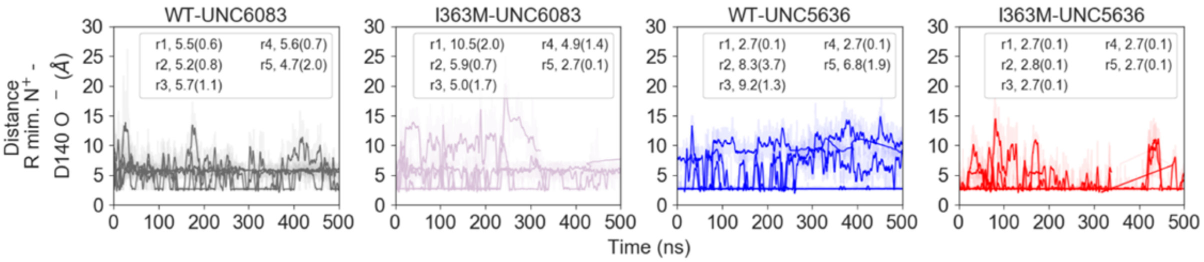

Supplementary Figure S11. Distances between N^{+} of arginine mimic of the ligand and O^{-} of D140 of EZH2 protein.

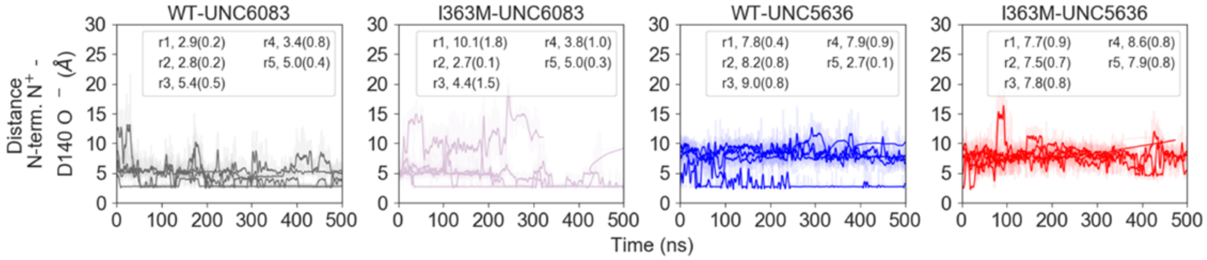

Supplementary Figure S12. Distance between ligand's R mimic N^+ and EED D362 O^-.

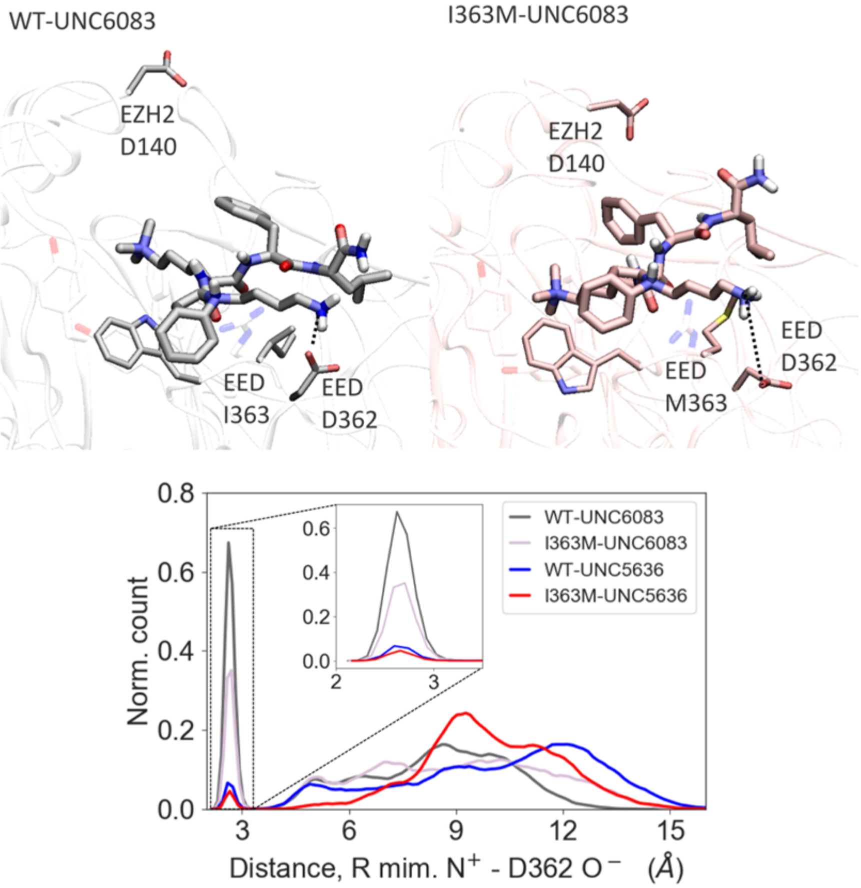

Supplementary Figure S13. Distance distributions between ligand's arginine mimic N^+ and EED D362 O^- (**SI Fig. S12**). Octahydroindole of **3** (UNC6083; inhibitor) prefers to lay 'flat' over the surface of EED protein and orient its arginine mimic towards and form a salt bridge with D362 of EED. Among the four simulated systems R mimic of compound **3** in wild type has the lowest tendency to form a salt bridge with D140 of EZH2 (**SI Fig. S11**), and the highest tendency to form a salt bridge with D362 of EED, which explains the experimental data indicating the highest degree of inhibition of the catalytic activity of the complex in WT-UNC6083 system. Since the simulations started from a hypothetical pose of the ligand in which its arginine mimic forms a salt bridge with EZH2 D140 and we used all collected data to plot these distributions, the distributions are skewed towards the higher values of the distance. However, the zoomed-in plot (insert) clearly indicates that the states possessing this salt bridge have started to be populated and the ranking order based on this distance distribution clearly

explains the experimental findings of the inhibition of the complex. Additionally, this data explains the lowest RMSD of the ligand (**SI Fig. S14**) in WT-UNC6083 system—the formation of this salt bridge ‘locks’ the molecule on the surface of EED, which could also indicate a higher affinity of the ligand to wild type EED.

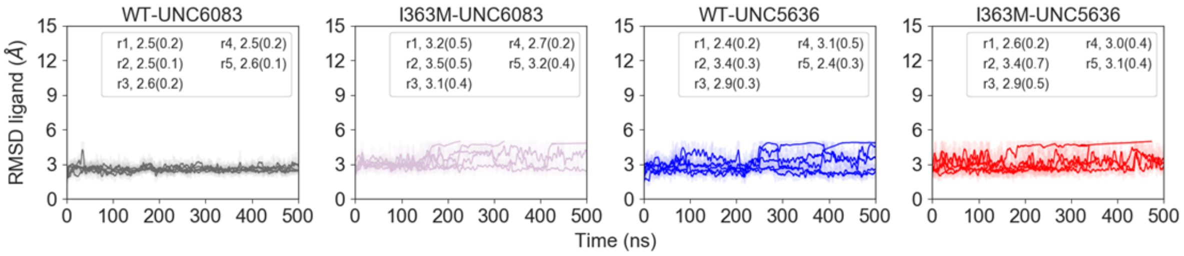

Supplementary Figure S14. RMSD of the ligand upon EED Cα alignment

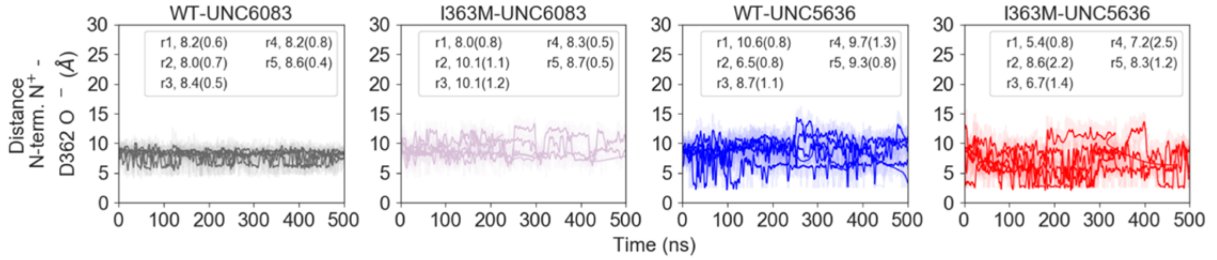

Supplementary Figure S15. Distance between ligand’s N-terminus N^+ and EED D362 O^-.

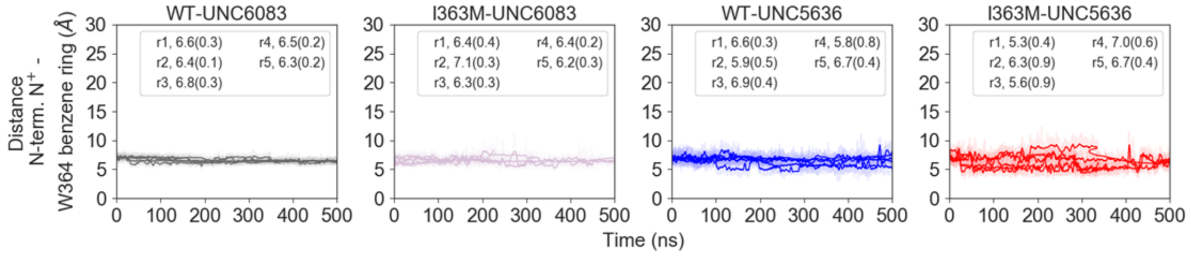

Supplementary Figure S16. Distance between ligand’s N-terminus N^+ and EED W364 benzene ring.

General chemistry procedures

Reverse phase column chromatography was performed with a Teledyne Isco CombiFlash®R_f 200 using C18 RediSep®R_f Gold columns with the UV detector set to 220 nm and 254 nm. Mobile phases of A (H_2O + 0.1% TFA) and B (MeOH or MeCN) were used with default column gradients. Preparative HPLC was performed using an Agilent Prep 1200 series with the UV detector set to 220 nm and 254 nm. Samples were injected onto either a Phenomenex Luna 250 × 30 mm (5 μm) C18 column or a Phenomenex Luna 75 x 30 mm (5 μm) C18 column at room temperature. Mobile phases of A (H_2O + 0.1% TFA) and B (MeOH or MeCN) were used with a flow rate of 40 mL/min for the large column and 30 mL/min for the small column. A general gradient of 0-22 minutes increasing from 10 to 100% B, followed by a 100% B flush for another 8 minutes. Small variations in this purification method were made as needed to achieve ideal separation for each compound.

Analysis of products

Analytical LCMS (at 220 nm and 254 nm) was used to establish the purity of targeted compounds. All compounds that were evaluated in biochemical and biophysical assays had >95% purity as determined by LCMS. Analytical LCMS data for all compounds were acquired using an Agilent 6110 Series system with the UV detector set to 220 nm and 254 nm. Samples were injected (<10 μL) onto an Agilent Eclipse Plus 4.6 × 50 mm, 1.8 μm, C18 column at room temperature. Mobile phases A (H_2O + 0.1% acetic acid) and B (MeOH + 0.1% acetic acid) were used with a linear gradient from 10% to 100% B in 5.0 min, followed by a flush at 100% B for another 2 minutes with a flow rate of 1.0 mL/min. Mass spectra (MS) data were acquired in positive ion mode using an Agilent 6110 single quadrupole mass spectrometer with an electrospray ionization (ESI) source. ^{1}H NMR spectra were obtained on a Varian automated 400MR at 400 MHz and 100 MHz. Chemical shifts are reported in ppm and coupling constants are reported in Hz with MeOD-d_4 referenced at 3.31 (^{1}H).

(S)-6-(((S)-1-(((S)-1-amino-4-methyl-1-oxopentan-2-yl)amino)-1-oxo-3-phenylpropan-2-yl)amino)-5-((S)-5-guanidino-2-((S)-pyrrolidine-2-carboxamido)pentanamido)-N,N,N-trimethyl-6-oxohexan-1-aminium (UNC5635, 1)

^{1}H-NMR (MeOD-d_4, 400 MHz): δ 8.67 (d, *J* = 7.2 Hz, backbone amide proton), 8.27 (d, *J* = 7.6 Hz, backbone amide proton), 8.18 (d, *J* = 5.2 Hz, backbone amide proton), 8.16 (d, *J* = 6.0 Hz, backbone amide proton), 7.31-7.19 (m, 5H), 4.66-4.63 (m, 1H), 4.38-4.28 (m, 4H), 3.42-3.28 (m, 2H), 3.29-3.16 (m, 4H), 3.13 (s, 9H), 2.97 (dd, *J* = 8.4 Hz, 14 Hz, 1H), 2.49-2.40 (m, 1H), 2.15-1.96 (m, 3H), 1.86-1.56 (m, 12H), 1.42-1.36 (m, 2H), (dd, *J* = 6.4 Hz, 17.2 Hz, 6H); MS (ESI): m/z calcd. for $[C_{35}H_{63}N_{10}O_5]^{2+}$ 351.75, found 351.35.

(S)-5-((S)-6-amino-2-((S)-pyrrolidine-2-carboxamido)hexanamido)-6-(((S)-1-(((S)-1-amino-4-methyl-1-oxopentan-2-yl)amino)-1-oxo-3-phenylpropan-2-yl)amino)-N,N,N-trimethyl-6-oxohexan-1-aminium (UNC5636, 2)

^{1}H-NMR (MeOD-d_4, 400 MHz): δ 8.26 (d, *J* = 7.6 Hz, backbone amide proton), 8.18 (d, *J* = 4.8 Hz, backbone amide proton), 8.16 (d, *J* = 6.4 Hz, backbone amide proton), 7.31-7.20 (m, 5H), 4.65-4.62 (m, 1H), 4.35-4.28 (m, 4H), 3.42-3.28 (m, 3fzH), 3.17-3.15 (m, 1H), 3.13 (s, 9H), 3.01-2.97 (m, 1H), 2.95-2.91 (m, 2H), 2.48-2.40 (m, 1H), 2.14-1.96 (m, 3H), 1.83-1.36 (m, 16H), 0.95-0.89 (dd, *J* = 6.4 Hz, 17.2 Hz, 6H); MS (ESI): m/z calcd. for $[C_{35}H_{63}N_8O_5]^{2+}$ 337.75, found 337.35.

(5S)-6-(((S)-1-(((S)-1-amino-4-methyl-1-oxopentan-2-yl)amino)-1-oxo-3-phenylpropan-2-yl)amino)-5-((3aS,7aS)-1-(4-aminobutyl)octahydro-1H-indole-2-carboxamido)-N,N,N-trimethyl-6-oxohexan-1-aminium (UNC6083, 3)

^{1}H-NMR (MeOD-d_4, 400 MHz): δ 8.18 (d, *J* = 8.0 Hz, backbone amide proton), 7.29-7.21 (m, 5H), 4.69-4.65 (m, 1H), 4.43-4.29 (m, 2H), 3.69-3.58 (m, 1H), 3.37-3.35 (m, 2H), 3.20-3.16 (m, 2H), 3.14 (s, 9H), 3.01-2.95 (m, 3H), 2.66-2.63 (m, 1H), 2.53-2.49 (m, 1H), 2.16-2.13 (m, 1H), 2.04-1.95 (m, 1H), 1.83-1.28 (m, 22H), 0.95-0.89 (dd, *J* = 6.4 Hz, 17.2 Hz, 6H); MS (ESI): m/z calcd. for $[C_{37}H_{66}N_7O_4]^{2+}$ 336.26, found 335.80.

(S)-5-((S)-6-amino-2-((S)-1-methylpyrrolidine-2-carboxamido)hexanamido)-6-(((S)-1-(((S)-1-amino-4-methyl-1-oxopentan-2-yl)amino)-1-oxo-3-phenylpropan-2-yl)amino)-N,N,N-trimethyl-6-oxohexan-1-aminium (UNC6008, 4)

^{1}H-NMR (MeOD-d_4, 400 MHz): δ 8.30 (d, *J* = 7.6 Hz, backbone amide proton), 8.18 (t, *J* = 8.0 Hz, backbone amide proton), 7.31-7.20 (m, 5H), 4.65-4.62 (m, 1H), 4.36-4.28 (m, 3H), 4.18-4.14

(t, J = 8.0 Hz, 1H), 3.72-3.69 (m, 1H), 3.35-3.28 (m, 1H), 3.25-3.20 (m, 1H), 3.17-3.12 (m, 10H), 3.01-2.91 (m, 6H), 2.64-2.55 (m, 1H), 2.23-1.96 (m, 3H), 1.87-1.28 (m, 16H), 0.95-0.89 (dd, J = 6.4 Hz, 17.2 Hz, 6H); MS (ESI): m/z calcd. for $[C_{36}H_{65}N_8O_5]^{2+}$ 344.75, found 344.30.

(S)-5-((S)-2-((S)-1-acetylpyrrolidine-2-carboxamido)-6-aminohexanamido)-6-(((S)-1-(((S)-1-amino-4-methyl-1-oxopentan-2-yl)amino)-1-oxo-3-phenylpropan-2-yl)amino)-N,N,N-trimethyl-6-oxohexan-1-aminium (UNC6009, 5)

^{1}H-NMR (MeOD-d_4, 400 MHz): δ 8.34 (d, J = 7.6 Hz, backbone amide proton), 8.07-8.02 (m, backbone amide proton), 7.31-7.19 (m, 5H), 4.62-4.58 (m, 1H), 4.37-4.24 (m, 4H), 3.71-3.52 (m, 2H), 3.34-3.25 (m, 2H), 3.20-3.15 (m, 1H), 3.12 (s, 9H), 3.01-2.91 (m, 3H), 2.39-2.22 (m, 1H), 2.11 (s, 3H), 2.07-2.01 (m, 1H), 1.98-1.95 (m, 1H), 1.88-1.57 (m, 12H), 1.56-1.42 (m, 2H), 1.37-1.29 (m, 2H), 0.95-0.89 (dd, J = 6.4 Hz, 17.2 Hz, 6H); MS (ESI): m/z calcd. for $[C_{37}H_{65}N_8O_6]^{2+}$ 358.75, found 358.30.

(5S)-6-(((S)-1-(((S)-1-amino-4-methyl-1-oxopentan-2-yl)amino)-1-oxo-3-phenylpropan-2-yl)amino)-5-((3aS,7aS)-1-(2-aminoethyl)octahydro-1H-indole-2-carboxamido)-N,N,N-trimethyl-6-oxohexan-1-aminium (UNC6081, 6)

^{1}H-NMR (MeOD-d_4, 400 MHz): δ 8.17 (d, J = 8.0 Hz, backbone amide proton), 7.31-7.20 (m, 5H), 4.67-4.63 (m, 1H), 4.37-4.31 (m, 2H), 3.63-3.58 (m, 1H), 3.37-3.33 (m, 1H), 3.23-2.83 (m, 15H), 2.45-2.38 (m, 1H), 2.23-2.13 (m, 1H), 1.88-1.28 (m, 20H), 0.95-0.89 (dd, J = 6.4 Hz, 17.2 Hz, 6H); MS (ESI): m/z calcd. for $[C_{35}H_{62}N_7O_4]^{2+}$ 322.24, found 321.80.

(5S)-6-(((S)-1-(((S)-1-amino-4-methyl-1-oxopentan-2-yl)amino)-1-oxo-3-phenylpropan-2-yl)amino)-5-((3aS,7aS)-1-(3-aminopropyl)octahydro-1H-indole-2-carboxamido)-N,N,N-trimethyl-6-oxohexan-1-aminium (UNC6082, 7)

^{1}H-NMR (MeOD-d_4, 400 MHz): δ 8.21 (t, J = 8.0 Hz, backbone amide proton), 7.31-7.19 (m, 5H), 4.69-4.65 (m, 1H), 4.42-4.28 (m, 2H), 3.68-3.58 (m, 1H), 3.42-3.14 (m, 13H), 3.01-2.95 (m, 3H), 2.66-2.62 (m, 1H), 2.55-2.48 (m, 1H), 2.18-1.97 (m, 4H), 1.86-1.28 (m, 18H), 0.95-0.89 (dd, J = 6.4 Hz, 17.2 Hz, 6H); MS (ESI): m/z calcd. for $[C_{36}H_{64}N_7O_4]^{2+}$ 329.25, found 328.90.

(9-(4-((3-(5-((12S,15S,18S)-18-((S)-6-amino-2-((S)-pyrrolidine-2-carboxamido)hexanamido)-2-(2-amino-2-oxoethyl)-15-benzyl-12-isobutyl-23,23-dimethyl-3,7,11,14,17-pentaoxo-2,6,10,13,16,23-hexaazatetracosan-23-ium-1-yl)-1H-1,2,3-triazol-1-yl)propyl)carbamoyl)-2-carboxyphenyl)-6-hydroxy-9H-xanthen-3-olate (UNC6419, 8, FAM-UNC5636)

The alkynyl peptoid was synthesized on Fmoc Rink amide MBHA resin (500 mg, 0.43 mmol/g loading, Anaspec). The alkynyl peptoid was installed as the first residue by coupling on bromoacetic acid (1 M) with N,N-diisopropylcarbodiimide (1 M) for 30 minutes followed by 7 DMF washes. Next, the displacement reaction was conducted with propargylamine (1 M) in DMF for 2 hours followed by 7 DMF washes. Following installation of the peptoid residue, standard Fmoc synthesis (as described in the solid-phase peptide synthesis section above) was used for the remaining residues. The alkynyl peptoid was then used to generate the labeled peptide through click chemistry with azido-fluorescein (1 eq, Tenova Pharmaceuticals), using copper sulfate (10 eq) and ascorbic acid (10 eq) dissolved in 1 mL DMF. The mixture was first heated to 70°C while stirring for 10 mins, after which the reaction was allowed to cool to room temperature and stirred overnight prior to concentration under vacuum. The crude mixture were re-dissolved in 1:1 water:acetonitrile (1 mL), filtered, and purified via preparative HPLC. Product fractions as determined by LCMS were concentrated under vacuum. Products were re-dissolved in water and lyophilized to yield a yellow powder. Purity was determined via LCMS UV trace to be >95%. ^{1}H-NMR (MeOD-d_4, 400 MHz): δ 8.42 (s, backbone amide proton), 8.27 (d, J = 7.6 Hz, backbone amide proton), 8.20 (d, J = 7.6, backbone amide proton), 8.14-8.08 (m, 3H), 7.62 (s, backbone amide proton), 7.32-7.19 (m, 6H), 6.72 (d, J = 2.0 Hz, 2H), 6.64-6.54 (m, 4H), 4.65-4.01 (m, 10H), 3.48-3.27 (m, 9H), 3.12 (s, 9H), 3.01-2.91 (m, 3H), 2.86-2.76 (m, 1H), 2.66-2.53 (m, 1H), 2.46-2.28 (m, 4H), 2.15-1.94 (m, 5H), 1.79-1.56 (m, 12H), 1.48-1.29 (m, 6H), 0.92-0.85 (dd, J = 6.4 Hz, 17.2 Hz, 6H); MS (ESI): m/z calcd. for $[C_{70}H_{95}N_{15}O_{14}]^+$ 1369.72, found 1369.63.

N2-(((9H-fluoren-9-yl)methoxy)carbonyl)-N6,N6-dimethyl-L-lysine

N2-(((9H-fluoren-9-yl)methoxy)carbonyl)-N6,N6-dimethyl-L-lysine was synthesized as previously reported (1). Fmoc-Lys-OH (3 g, 1 eq, 8 mmol) was dissolved in acetonitrile (20 mL). Formaldehyde (37 % w/v solution in water; 4.7 mL, 8 eq, 64 mmol) was added to the cloudy

solution while stirring at room temperature. After 5 min, acetic acid (2 mL, 5 eq, 40 mmol) and sodium cyanoborohydride (0.6 g, 2 eq, 16 mmol) were added to the reaction and the mixture was left to stir at room temperature for 2 hours. The reaction was concentrated *in vacuo* and re-dissolved in 1:1 water:acetonitrile (10 mL). The crude material was purified via reverse phase flash chromatography (H2O + 0.1% trifluoroacetic acid and acetonitrile) to yield product. The product was lyophilized to a hygroscopic, white powder and yielded 2.52 g (80%) of product.

N2-(((9H-fluoren-9-yl)methoxy)carbonyl)-N6,N6,N6-trimethyl-L-lysine

N2-(((9H-fluoren-9-yl)methoxy)carbonyl)-N6,N6-dimethyl-L-lysine (TFA salt) (1.8 g, 1 eq, 4.5 mmol) was dissolved in acetonitrile (3 mL) and water (10 mL). Potassium carbonate (0.82 g, 1.3 eq, 5.9 mmol) was added while stirring at room temperature. Methyl iodide (2.8 mL, 10 eq, 45 mmol) was added to the solution and the mixture was left to stir overnight at room temperature. The reaction was concentrated *in vacuo* and re-dissolved in 1:1 water:acetonitrile (10 mL). The crude material was purified via reverse phase flash chromatography (H_2O + 0.1% trifluoroacetic acid and acetonitrile) to yield product. The product was lyophilized to a hygroscopic, white powder and yielded 1 g (50%) of product. ^{1}H NMR (MeOD-d_4, 400 MHz): δ 7.82 (d, J = 7.2 Hz, 2H), 7.70 (t, J = 8.4 Hz, 2H), 7.42 (t, J = 7.6 Hz, 2H), 7.34 (td, J = 1.2 Hz, 7.6 Hz, 2H), 4.45-4.40 (m, 1H), 4.35-4.31 (m, 1H), 4.25-4.19 (m, 2H), 3.36 – 3.25 (m, 2H), 3.11 (s, 9H), 2.00-1.92 (m, 1H), 1.84-1.72 (m, 3H), 1.49-1.43 (m, 2H); MS (ESI): m/z calcd. for $[C_{24}H_{31}N_2O_4]^+$ 411.23, found 411.30.

^{1}H NMR Spectra

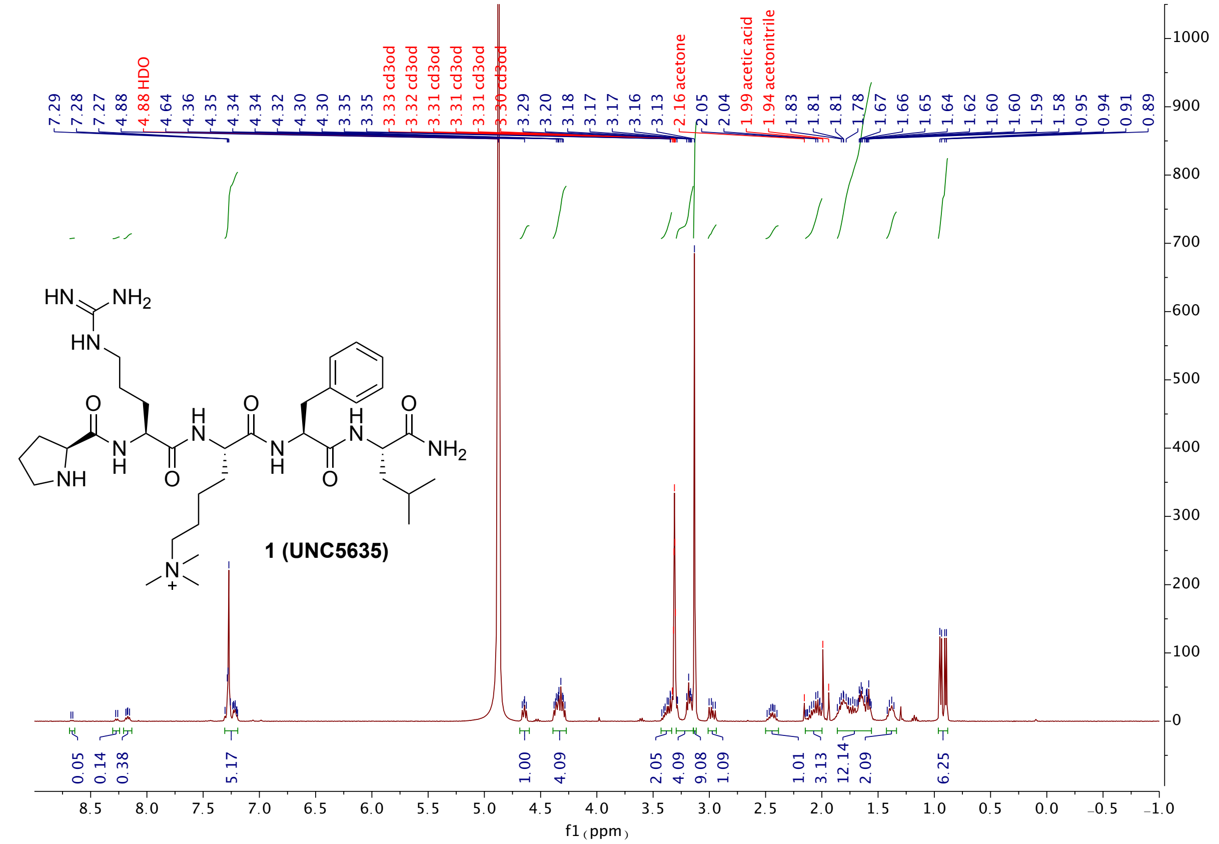

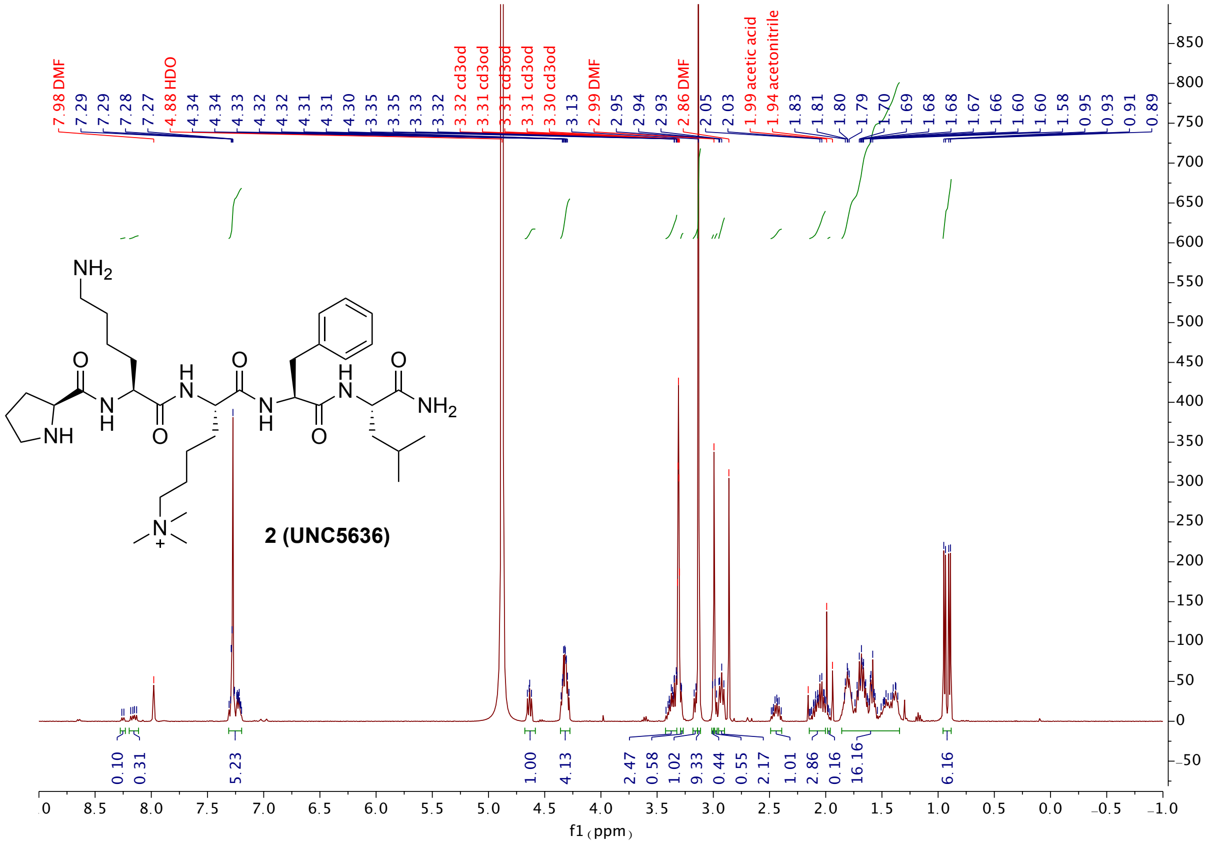

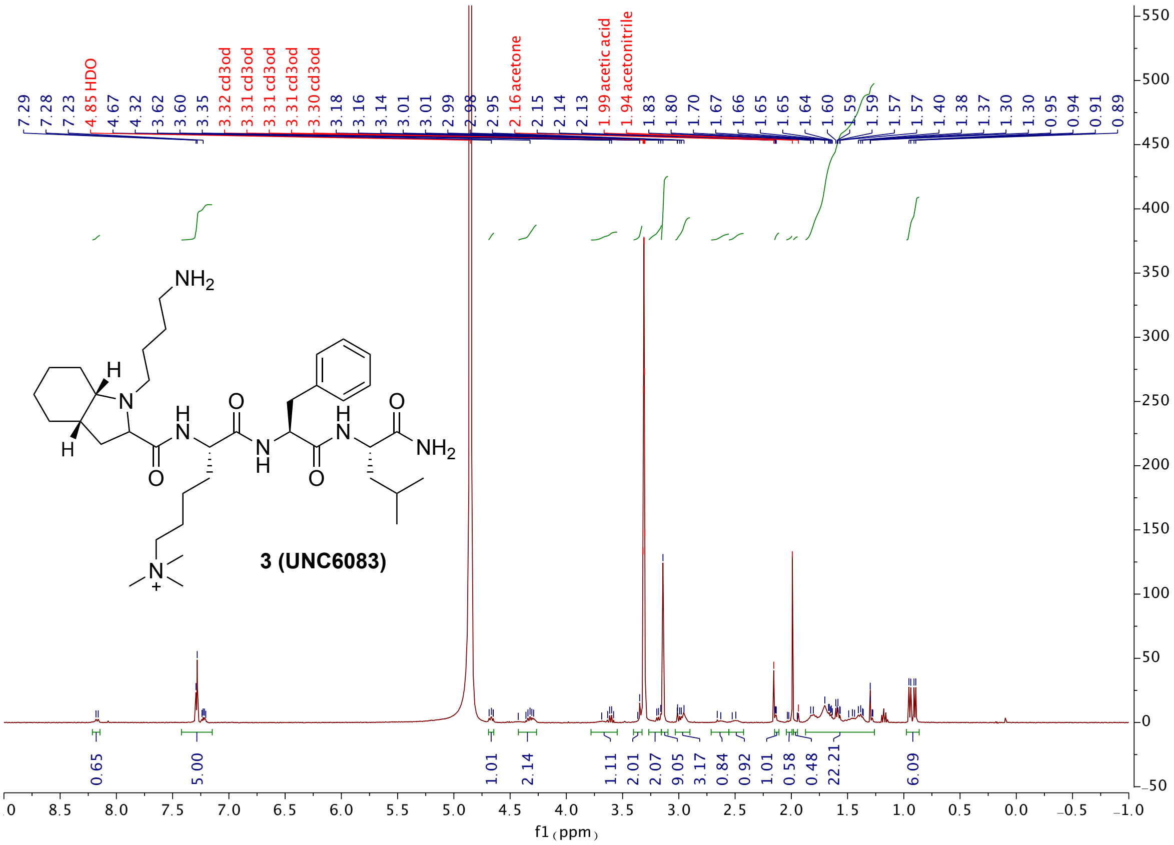
3 (UNC6083)
f1 (ppm)

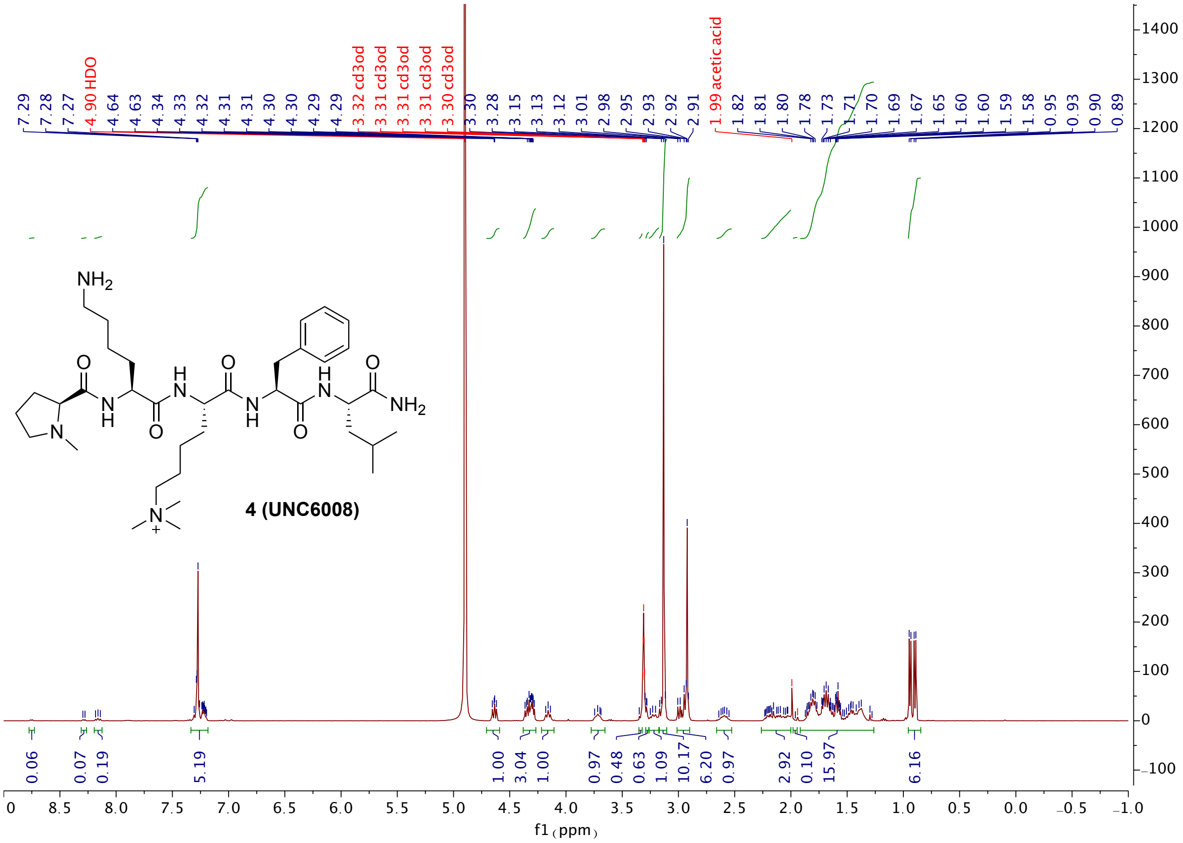
4 (UNC6008)
f1 (ppm)

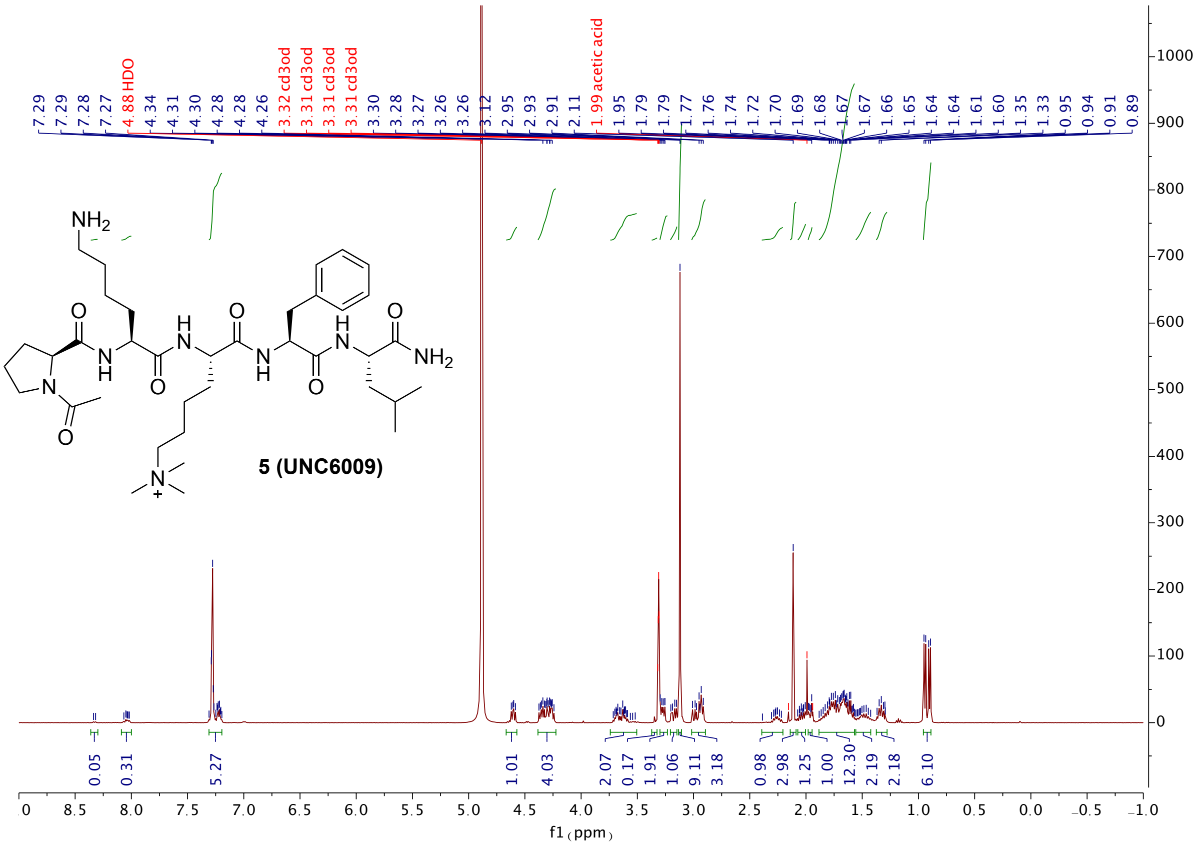
7.29 7.29 7.28 7.27 4.88 HDO 4.34 4.31 4.30 4.28 4.28 4.26 3.32 cd3od 3.31 cd3od 3.31 cd3od 3.31 cd3od 3.30 3.28 3.27 3.26 3.26 3.12 2.95 2.93 2.91 2.11 1.99 acetic acid 1.95 1.79 1.79 1.77 1.76 1.74 1.72 1.70 1.69 1.68 1.67 1.67 1.66 1.65 1.64 1.64 1.61 1.60 1.35 1.33 0.95 0.94 0.91 0.89
NH2
5 (UNC6009)
0.05 0.31 5.27 1.01 4.03 2.07 0.17 1.91 1.06 9.11 3.18 0.98 2.98 1.25 1.00 12.30 2.19 2.18 6.10
f1 (ppm)

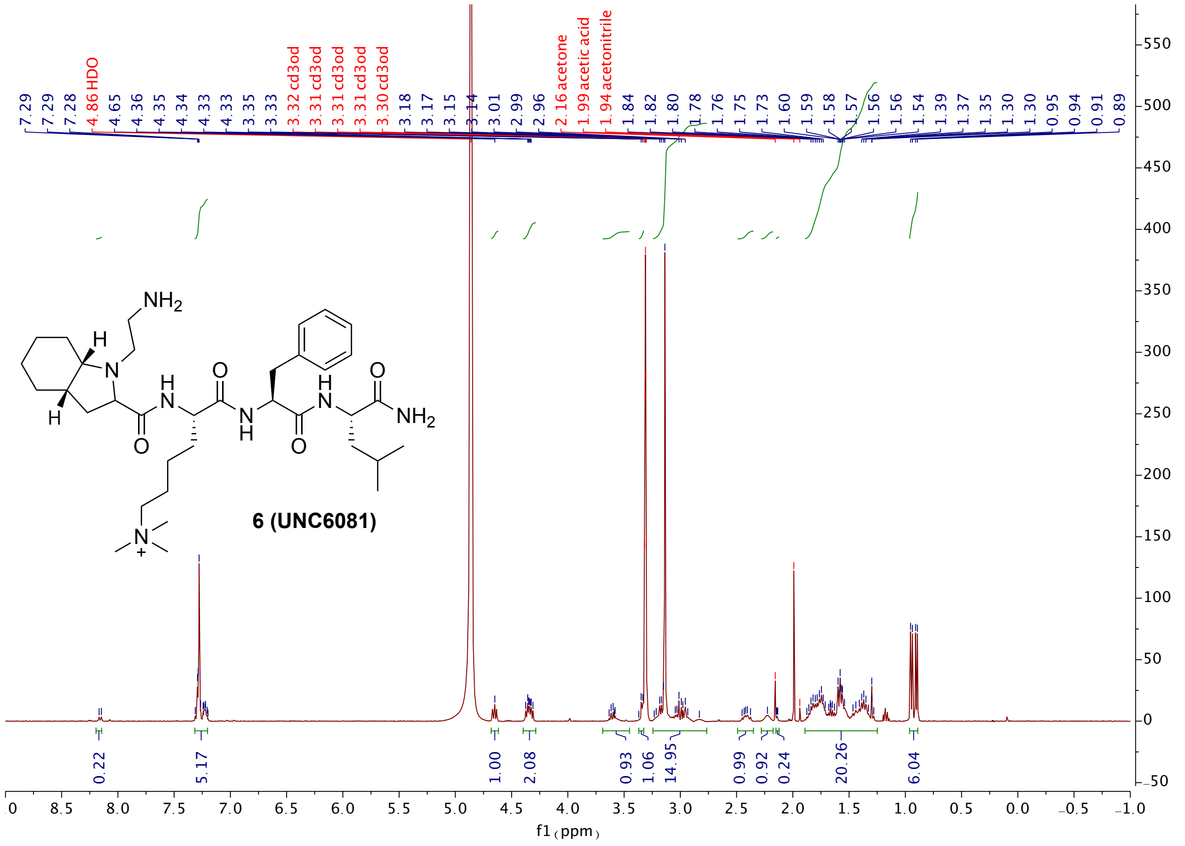
7.29 7.29 7.28 4.86 HDO 4.65 4.36 4.35 4.34 4.33 4.33 3.35 3.33 3.32 cd3od 3.31 cd3od 3.31 cd3od 3.31 cd3od 3.30 cd3od 3.18 3.17 3.15 3.14 3.01 2.99 2.96 2.16 acetone 1.99 acetic acid 1.94 acetonitrile 1.84 1.82 1.80 1.78 1.76 1.75 1.73 1.60 1.59 1.58 1.57 1.56 1.54 1.39 1.37 1.35 1.30 0.95 0.94 0.91 0.89
NH2
6 (UNC6081)
0.22 5.17 1.00 2.08 0.93 1.06 14.95 0.99 0.92 0.24 20.26 6.04
f1 (ppm)

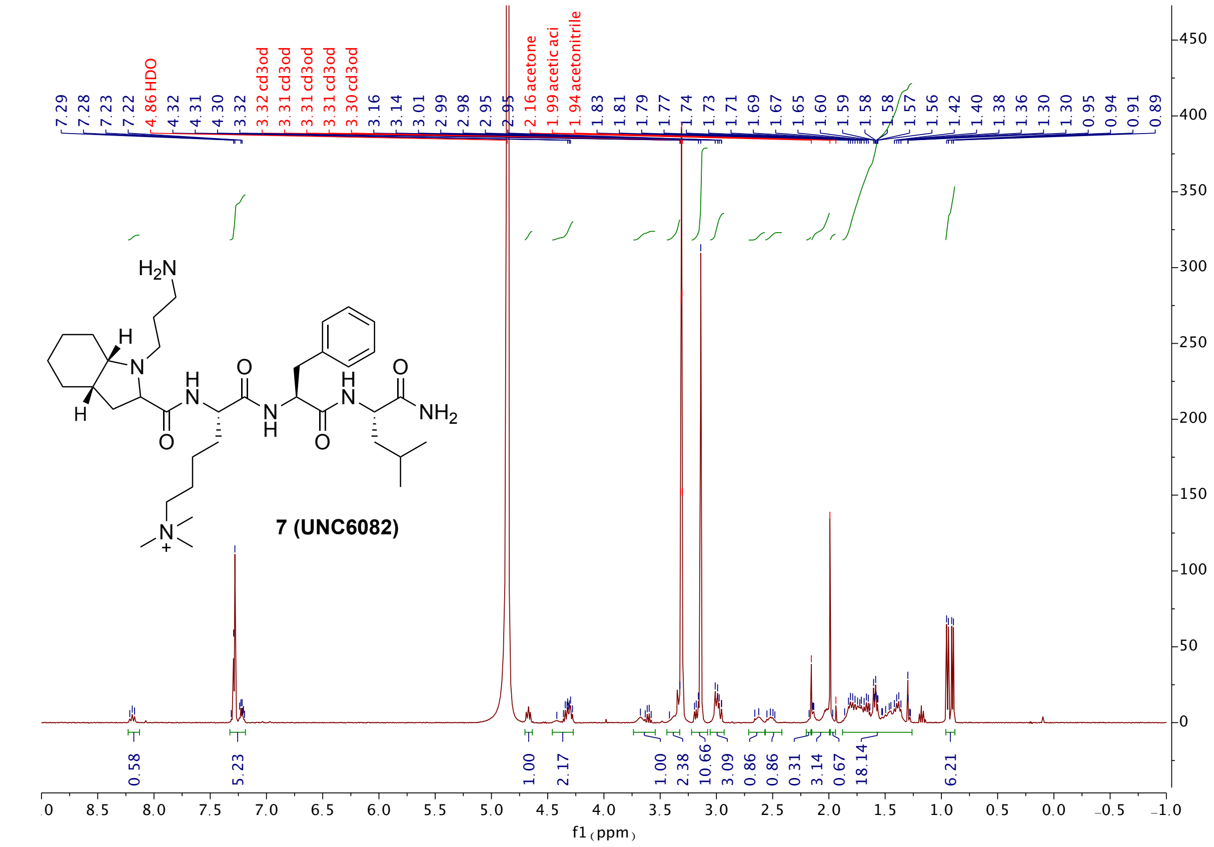
7 (UNC6082)
7.29 7.28 7.23 7.22 4.86 HDO 4.32 4.31 4.30 3.32 3.32 cd3od 3.31 cd3od 3.31 cd3od 3.31 cd3od 3.30 cd3od 3.16 3.14 3.01 2.99 2.98 2.95 2.95 2.16 acetone 1.99 acetic aci 1.94 acetonitrile 1.83 1.81 1.79 1.77 1.74 1.73 1.71 1.69 1.67 1.65 1.60 1.59 1.58 1.58 1.57 1.56 1.42 1.40 1.38 1.36 1.30 0.95 0.94 0.91 0.89
0.58 5.23 1.00 2.17 1.00 2.38 10.66 3.09 0.86 0.86 0.31 3.14 0.67 18.14 6.21
f1 (ppm)

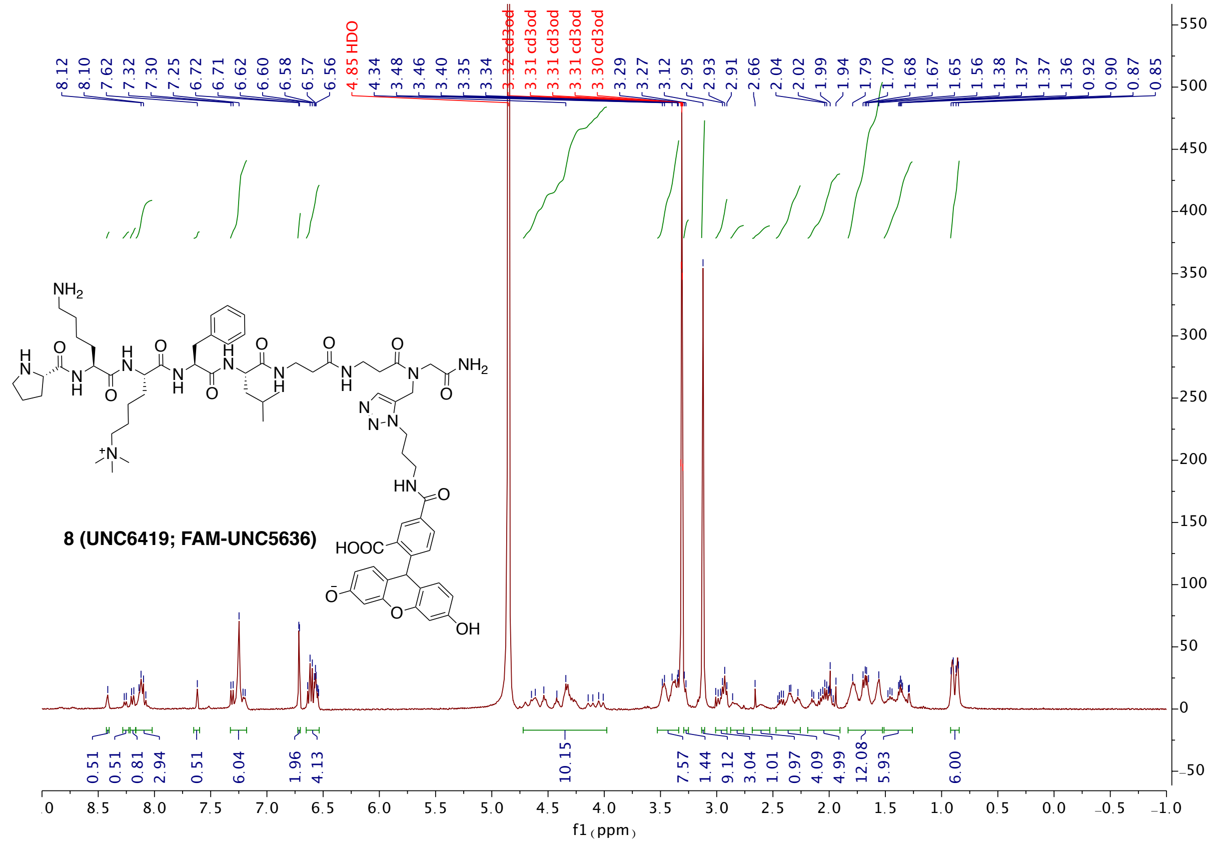
8 (UNC6419; FAM-UNC5636)
8.12 8.10 7.62 7.32 7.30 7.25 6.72 6.71 6.62 6.60 6.58 6.57 6.56 4.85 HDO 4.34 3.48 3.46 3.40 3.35 3.34 3.32 cd3od 3.31 cd3od 3.31 cd3od 3.31 cd3od 3.30 cd3od 3.29 3.27 3.12 2.95 2.93 2.91 2.66 2.04 2.02 1.99 1.94 1.79 1.70 1.68 1.67 1.65 1.56 1.38 1.37 1.37 1.36 0.92 0.90 0.87 0.85
0.51 0.51 0.81 2.94 0.51 6.04 1.96 4.13 10.15 7.57 1.44 9.12 3.04 1.01 0.97 4.09 4.99 12.08 5.93 6.00
f1 (ppm)

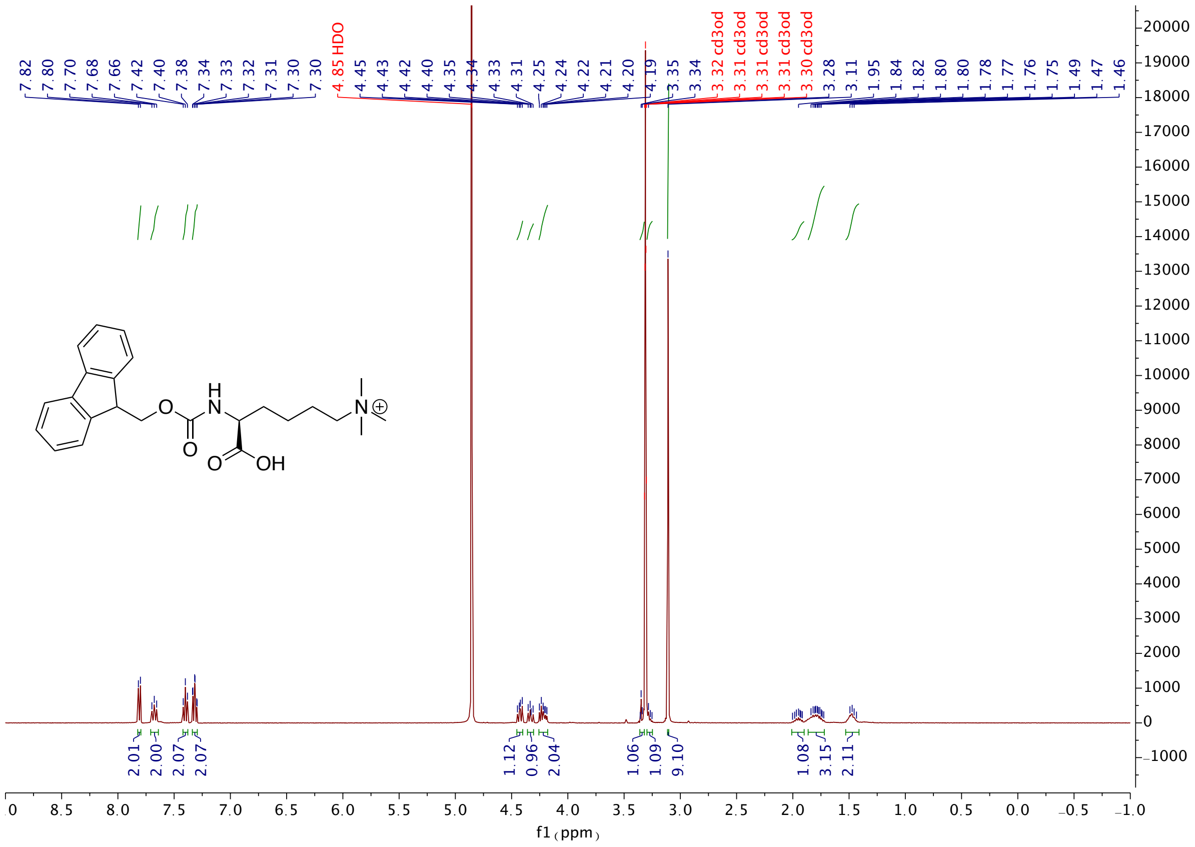

Supplementary Movies

Movie S1. Interactions of compound **2** with WT PRC2. In WT PRC2, compound **2** is able to form interactions with the EZH2 loop, however, these contacts are significantly less frequent than in the mutant complex, thereby preventing effective stabilization of the SRM helix, which has been previously shown to be a key component of the EZH2 catalytically active state (see the main manuscript for details). The color scheme is the same as that in Figure 4.

Movie S2. Interactions of compound **2** with PRC2 EED-I363M mutant. The most distinctive feature of the dynamic behavior of compound **2** in PRC2-EED-I363M is the persistent ionic interactions between the unmodified lysine side chain of the compound and D140 located in a flexible loop of EZH2 (residues 136-142), which drastically reduces the mobility of the loop, thus preserving the integrity of the adjacent SRM helix (see the main manuscript for details). The color scheme is the same as that in Figure 4.

References

1. Barnash, K. D., The, J., Norris-Drouin, J. L., Cholensky, S. H., Worley, B. M., Li, F. L., Stuckey, J. I., Brown, P. J., Vedadi, M., Arrowsmith, C. H., Frye, S. V., and James, L. I. (2017) Discovery of Peptidomimetic Ligands of EED as Allosteric Inhibitors of PRC2. *Acs Comb Sci* **19**, 161-172
2. Justin, N., Zhang, Y., Tarricone, C., Martin, S. R., Chen, S., Underwood, E., De Marco, V., Haire, L. F., Walker, P. A., Reinberg, D., Wilson, J. R., and Gamblin, S. J. (2016) Structural basis of oncogenic histone H3K27M inhibition of human polycomb repressive complex 2. *Nat Commun* **7**, 11316
